# A Cell-Based Optimised Approach for Rapid and Efficient Gene Editing of Human Pluripotent Stem Cells

**DOI:** 10.3390/ijms241210266

**Published:** 2023-06-17

**Authors:** Sara Cuevas-Ocaña, Jin Ye Yang, Magomet Aushev, George Schlossmacher, Christine E. Bear, Nicholas R. F. Hannan, Neil D. Perkins, Janet Rossant, Amy P. Wong, Michael A. Gray

**Affiliations:** 1 Biosciences Institute, Medical School, Newcastle University, Newcastle upon Tyne NE2 4HH, UK; george.schlossmacher@biosynth.com (G.S.); neil.perkins@newcastle.ac.uk (N.D.P.); m.a.gray@ncl.ac.uk (M.A.G.); 2 Biodiscovery Institute, Translational Medical Sciences, School of Medicine, University of Nottingham, Nottingham NG7 2RD, UK; nick.hannan@nottingham.ac.uk; 3 Programme in Developmental & Stem Cell Biology, Peter Gilgan Centre for Research and Learning, The Hospital for Sick Children, Toronto, ON M5G 0A4, Canada; joeyyang91@hotmail.com (J.Y.Y.); janet.rossant@sickkids.ca (J.R.); apwong@sickkids.ca (A.P.W.); 4 Wellcome Trust Centre for Mitochondrial Research, Institute of Genetic Medicine, Biomedicine West Wing, Centre for Life, Times Square, Newcastle upon Tyne NE1 3BZ, UK; mag.aushev@newcastle.ac.uk; 5 Programme in Molecular Medicine, Peter Gilgan Centre for Research and Learning, The Hospital for Sick Children, Toronto, ON M5G 0A4, Canada; bear@sickkids.ca

**Keywords:** stem cells, gene editing, TALEN, CRISPR-Cas9, ΔF508, W1282X, cystic fibrosis

## Abstract

Introducing or correcting disease-causing mutations through genome editing in human pluripotent stem cells (hPSCs) followed by tissue-specific differentiation provide sustainable models of multiorgan diseases, such as cystic fibrosis (CF). However, low editing efficiency resulting in extended cell culture periods and the use of specialised equipment for fluorescence activated cell sorting (FACS) make hPSC genome editing still challenging. We aimed to investigate whether a combination of cell cycle synchronisation, single-stranded oligodeoxyribonucleotides, transient selection, manual clonal isolation, and rapid screening can improve the generation of correctly modified hPSCs. Here, we introduced the most common CF mutation, ΔF508, into the *CFTR* gene, using TALENs into hPSCs, and corrected the W1282X mutation using CRISPR-Cas9, in human-induced PSCs. This relatively simple method achieved up to 10% efficiency without the need for FACS, generating heterozygous and homozygous gene edited hPSCs within 3–6 weeks in order to understand genetic determinants of disease and precision medicine.

## 1. Introduction

The use of gene editing technologies is changing the way we approach research and medicine. Zinc-finger nucleases (ZFNs) [1], transcription activator-like effector nucleases (TALENs) [2,3], and clustered regularly interspaced short palindromic repeats (CRISPR)-Cas9 [4,5] have been widely used to model genetic diseases by enabling the precise introduction of any modification in the genome [6]. Although more recently described base [7,8] and prime editors [9] might provide additional advantages, both TALENs and CRISPR-Cas9 editing tools are still widely used. These technologies target specific DNA regions and introduce nuclease-mediated double-strand breaks (DSBs). DSBs can be repaired by the non-homologous end-joining (NHEJ) pathway usually introducing indels, small insertions and deletions, at the target site [10]. In the presence of a DNA template, DSBs instead activate the homology-directed repair (HDR) pathway, enabling the introduction of precise modifications in the genome [3].

TALENs and CRISPR-Cas9-mediated genome editing has enabled the development of numerous in vitro models by introducing de novo mutations into wild-type (WT) cells or by correcting specific mutations present in patient-derived cells. Both approaches generate powerful isogenic controls that have opened new avenues for regenerative medicine and personalised drug-testing applications [11]. When applied to human pluripotent stem cells (hPSCs), gene editing technologies, in combination with refined differentiation protocols, generate unique sources of cells for modelling multiorgan diseases.

For multiorgan disorders, such as cystic fibrosis (CF), gene edited hPSCs enable unlimited production of in vitro models potentially for every affected organ, including the lungs [12], from the same initial source of hPSCs, without the need for any patient material [13] or just one minimally invasive patient sample [14]. Genetically defined and patient-derived tissue-specific in vitro models of CF continue to be highly relevant to find suitable treatments for the remaining ~10–18% of people with CF who will not benefit from current CFTR-directed drug treatments [15,16,17], in order to assess personalised CFTR modulator responses and investigate non-lung CF symptoms [18,19,20].

Although feasible [10], gene editing of hPSCs using nuclease-based approaches remains challenging, mostly due to the low efficiency of generating precise genetic modifications through HDR in hPSCs [21]. This limitation is especially relevant when homozygous modifications are needed, most often requiring two rounds of targeting to achieve the desired changes. Additional rounds of targeting further increase the already high costs associated with the extended periods of hPSC culture required in most methods to obtain correctly edited hPSCs [22,23,24,25,26,27]. Efforts to overcome this low efficiency have focused on the use of fluorescence activated cell sorting (FACS) to select or enrich for targeted cells [6,28,29,30]. However, FACS cannot be applied to all hPSCs as it can compromise cell survival [28,31,32,33] and can induce stress-related spontaneous differentiation in sensitive hPSCs, and, when feasible, the use of FACS for clonal isolation still requires cell line-specific optimization [28].

The need for FACS, the time-consuming processes, and high costs associated with the maintenance of hPSCs, in addition to the intrinsic complexity of hPSC manipulation [34], still pose a limitation for the wider use of genome edited hPSCs. Here, we sought to develop a robust, rapid, and thus cost-effective method to generate stable gene edited hPSC lines for disease modelling amenable to TALENs and CRISPR-Cas9 editing tools. Here, several strategies were explored, including modulation of the cell cycle, efficient transfection of designer nucleases in combination with single-stranded donor templates, enrichment for transfected cells, manual clonal isolation, and direct allele-specific PCR (ASPCR) [35] screening. We used TALENs and CRISPR-Cas9 to target two different sites of the *CFTR* gene: (1) to introduce the most common CF mutation, ΔF508, and (2) to correct the rare nonsense mutation, W1282X, for CF modelling in human embryonic stem cells (hESCs) [36] and three patient-derived induced pluripotent stem cells (iPSCs) [37], respectively. This approach achieved relatively high editing efficiencies of 3–10%, eliminating the need for FACS, and generating gene edited hPSCs within 3–6 weeks. These results highlight the robustness and flexibility of this approach, amenable to different editing tools, which can potentially be applied to the new base [7,8] and prime editors [9], and be used for modelling other genetic diseases.

## 2. Results

### 2.1. CFTR-Specific TALEN Efficiently Modified hESCs Using Hypothermia and Enrichment for Transfected Cells

Transfection efficiency is a key factor that influences gene editing efficiency. An enhanced green fluorescent protein (eGFP)-encoding plasmid was nucleofected into the hESC line CA1 [13]. Optimal nucleofection of the CA1 cells was achieved using the B-016 program and Ingenio^®^ electroporation solution (Figure S1a,b). In addition, the CA1 cells were adapted to single-cell passaging conditions [38,39], which, together with nucleofection (Figure S1c) [40], did not compromise the expression of the pluripotency marker TRA-1-60 (Figure S2). This well-established CA1 hESC line was then used to introduce the most common CF-causing mutation, ΔF508, in order to generate an isogenic counterpart of CA1 hESCs for CF modelling.

**Figure 1:**
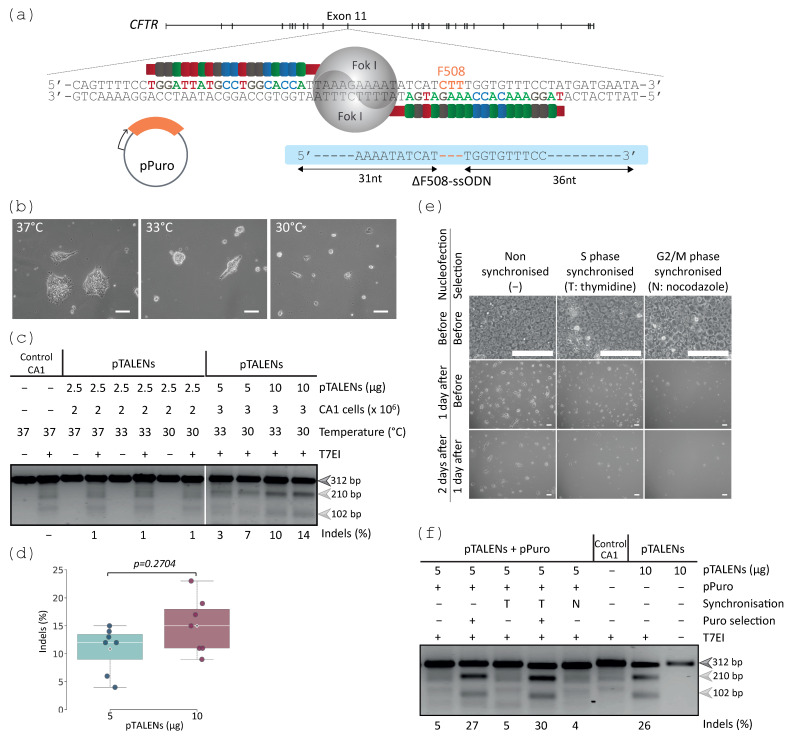
TALEN activity was detected under hypothermia and improved by transient puromycin selection. (**a**) Gene editing strategy to introduce the ΔF508 mutation (orange) into the CA1 cells (grey) using a TALEN pair (pTALENs) targeting exon 11 of *CFTR*, a ssODN harbouring ΔF508 (ΔF508-ssODN) and a plasmid containing puromycin resistant (pPuro). (**b**) Phase-contrast microscopy images of the CA1 cell populations (2 × 10^6^ CA1 cells) nucleofected with 2.5 µg pTALENs, 4 days after nucleofection and incubation under 37 °C, 33 °C, and 30 °C. Scale bars, 100 µm; (**c**) T7EI assay of the CA1 cultures nucleofected with pTALENs. Representative 2.5% agarose gel shows uncleaved amplicons (312 bp, dark grey arrow) and two cleavage products (210 bp and 102 bp, light grey arrows) confirming the correct TALEN activity. Control-untransfected CA1 cells. (**d**) Summary of TALEN activity from CA1 cell populations nucleofected with 5 µg (blue) or 10 µg (plum) pTALENs under 30 °C (n = 7 biological replicates per condition). Each data point represents the mean of TALEN activity from one nucleofected population, obtained from 1–5 repeated T7EI assays. The centre lines show the medians, crosses show the sample means, box limits indicate the quartiles with Tukey-whiskers extending 1.5 times the interquartile range. No significant difference was found in TALEN activity (Mann–Whitney U test). (**e**) Phase-contrast microscopy images of CA1 cell populations that were non synchronised, synchronised in S or G2/M phases of the cell cycle, prior to nucleofection of 3 × 10^6^ cells with 5 µg pTALENs and 2 µg pPuro, seeded in medium containing 10 µM Y-27632 and 1 µL/mL SCR7, incubated under 30 °C, and puromycin enriched for 3 days (0.7 µg/mL for 2 days and 1 µg/mL for 1 day). Scale bars, 100 µm. (**f**) The increase in indels (indicated by the 210 bp and 102 bp bands, light grey arrows, in 3% agarose gel) confirmed by the T7EI assay in these populations (**e**) suggests the enrichment of transfected cells by transient selection.

The gene editing strategy to introduce the ΔF508 mutation into CA1 cells consisted of a TALEN pair targeting a region in close proximity to F508 in exon 11 of *CFTR*, a single-stranded oligodeoxyribonucleotide (ssODN) harbouring ΔF508 as a donor template (Figure 1a) and an independent plasmid containing a puromycin resistance cassette (Figure 1a). ssODNs can be rapidly synthesised and this strategy would avoid the need for molecular cloning/construct generation. ssODNs have been used without impairing cell viability in combination with CRISPR-Cas9 in human stem cells [28] and with TALENs in ESCs [41], while achieving higher gene editing efficiencies compared with the double-stranded templates in combination with ZFNs [42].

CA1 cells were nucleofected with the engineered TALEN-encoding plasmids (pTALENs), followed by transient incubation under normothermia (37 °C), mild hypothermia (33 °C), or hypothermia (30 °C). Hypothermia was previously reported to increase the steady-state amount of nucleases proteins [43], improving nuclease activity [2,44,45,46], thus the aim was to assess whether this would improve TALEN activity in the CA1 cells. Although temperatures lower than 37 °C affected cell viability (Figure 1b), they did not compromise the pluripotency marker expression (Figure S2b). No measurable cleavage was detected by the T7 endonuclease I (T7EI) assay when using 2.5 µg pTALENs, but nucleofection of higher amounts of pTALENs and incubation under mild hypothermia or hypothermia confirmed the presence of indels at the target site within the CA1 populations (Figure 1c,d). Thus, hereafter, 5–10 µg pTALENs and 30 °C–33 °C incubation temperatures were adopted as optimal conditions for TALEN activity.

Previous reports have shown that cell cycle synchronisation was an effective approach to increase on-target NHEJ and HDR-mediated gene editing [47,48]. However, in most cases, the HDR efficiencies in hPSCs were still low, requiring sorting of the hPSCs [48] or treatments with several compounds [47]. Although conservative homologous recombination (HR) has been reported to repair DSBs at a higher frequency during the S phase in cancer-derived cells [49], and is correlated with high levels of DNA replication in mid-S phase [50], this strategy has not yet been largely explored to enhance gene editing efficiency in hPSCs. Based on previous studies, CA1 cells were synchronised in S or G2/M phases of the cell cycle by thymidine [51] and by nocodazole treatment [48], respectively, prior to nucleofection with pTALENs and pPuro. Cell culture density differed between populations after nucleofection under hypothermia (Figure 1e) with an observed decrease in cell viability in the G2/M phase synchronised populations. The analysis of indels performed by the T7EI assay revealed that transient puromycin enrichment can be applied to further increase the targeting efficiency in non-synchronised and S phase synchronised populations (5% versus 27% and 5% versus 30%, respectively) when lower amounts of plasmids are used to avoid compromising cell viability (Figure 1f). It was observed that G2/M phase synchronisation may negatively impact subsequent steps required for gene editing due to decreased cell viability.

### 2.2. Cell Cycle Synchronisation Prior to Nucleofection Was Tested for the Integration of the ΔF508 Mutation in hESCs

**Figure 2:**
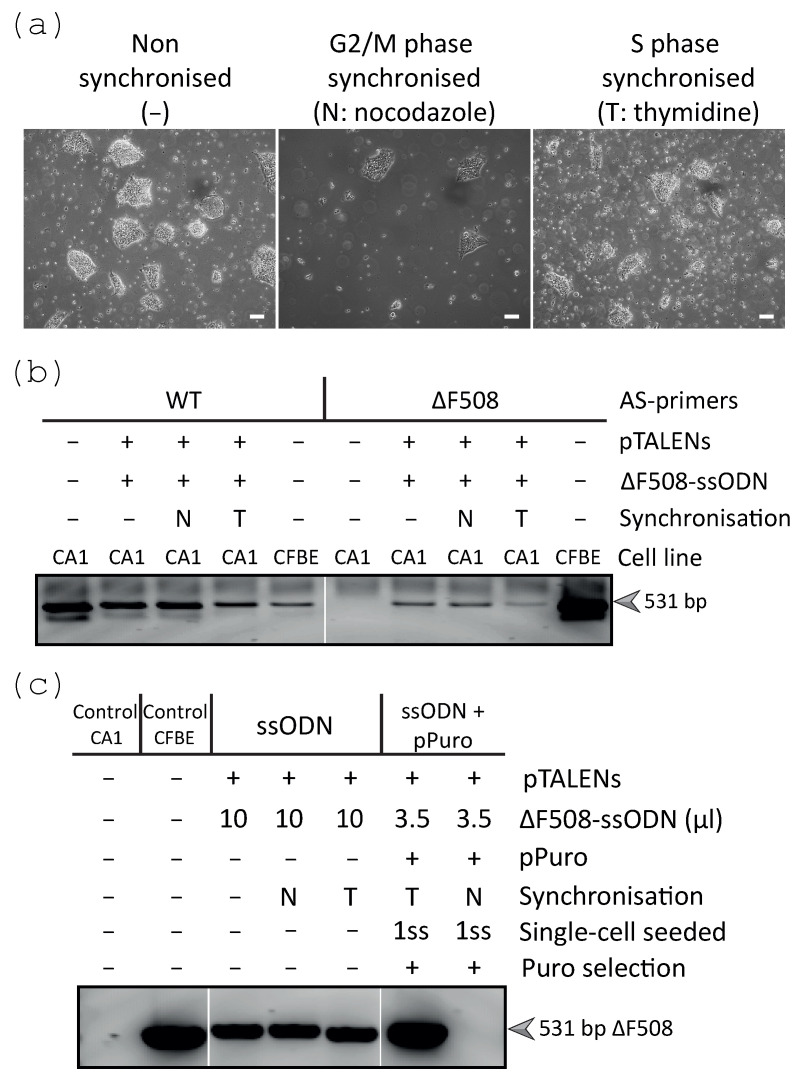
ASPCR ∆F508 indicated enhanced ΔF508 integration in the S phase synchronised CA1 cell population transfected with pTALENs, ΔF508-ssODN, and pPuro. (**a**) Phase-contrast microscopy images of the non-, G2/M, or S phase synchronised CA1 cell populations (3 × 10^6^ cells), 4 days after nucleofection with 5 µg pTALENs and 10 µL ΔF508-ssODN, seeded in medium containing 5 µM Y-27632 and 1 µL/mL SCR7, and incubated at 32 °C. Scale bars, 100 µm. (**b**) These populations (**a**) were assessed for the integration of ΔF508 by ASPCR, which detected unmodified (WT) or correctly modified sequences (ΔF508 mutation) as bands (531 bp arrow) in 1% agarose gel. Negative control-untransfected CA1 cells, positive control-CF bronchial epithelial cells (CFBE) expressing ΔF508. (**c**) CA1 cell populations (3 × 10^6^ cells) nucleofected with 5 µg pTALENs and ΔF508-ssODN, with or without 2 µg pPuro and transient selection (1 day at 0.7 µg/mL and 1 day at 1 µg/mL), seeded in medium containing 5 µM Y-27632 and incubated under 32 °C were also assessed by ASPCR. ΔF508 integration was detected by 1% agarose gel (531 bp arrow) in the described populations.

The pTALENs and the ΔF508 harbouring ssODN were nucleofected into non-synchronised, G2/M phase and S phase synchronised CA1 cell populations to assess HDR-mediated editing efficiency. Although cell density differed between populations, the surviving cells from all conditions were able to form colonies under mild hypothermia (32 °C) (Figure 2a). An allele-specific PCR (ASPCR) that relied on selective primers specifically amplifying either the WT or the ΔF508 alleles was optimised to detect the integration of the ΔF508 mutation (Figure 2b). A WT bronchial epithelial cell line stably transduced with ΔF508-*CFTR* (referred to as CFBE) was used as an inherent control for both WT and ΔF508. The presence of cells containing the ΔF508 mutation was confirmed by ASPCR within the three populations (Figure 2b). New S phase and G2/M phase synchronised populations were nucleofected with pTALENs, ΔF508-ssODN, and pPuro, followed by transient puromycin selection to enrich for transfected cells. The nucleofected populations (or a portion of them) could be cryopreserved before or after enrichment without compromising the total editing efficiency. Shortly after puromycin enrichment, when cultures started to form colonies, nucleofected populations were dissociated into single cells and reseeded within the same well, hereafter referred to as ‘1ss’, indicating **1** time dissociated into single cells and seeded after transfection. This dissociation marked the beginning of the clonal isolation process increasing the chances of isolating pure clones, and it also allowed for the collection of representative DNA from the populations to perform early population ASPCR screening. ASPCR confirmed the integration of the ΔF508 mutation within the nucleofected non-synchronised and S phase synchronised non-enriched and enriched populations, while it could not be detected in the G2/M phase synchronised enriched population, likely due to the described compromised cell viability (Figure 2c).

### 2.3. Clonal Expansion and Direct ASPCR Screening Enabled the Isolation of a ΔF508 Clone

**Figure 3:**
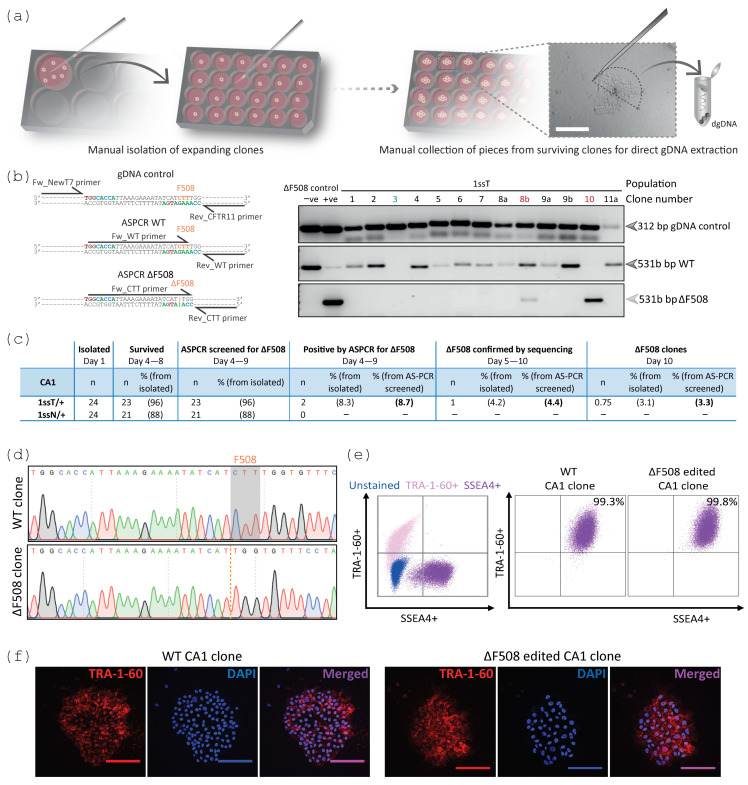
Direct ASPCR screening identified a potential homozygous ΔF508 clone from the S phase synchronised 1ss population, which retained the expression of pluripotency markers. (**a**) Clonal isolation was manually performed and shortly after, a small portion of each colony(s) of approximately 100 × 150 μm (grey shape) was manually sectioned and collected for direct gDNA (dgDNA) extraction. Scale bar, 100 μm. (**b**) This dgDNA was directly used for rapid clonal ASPCR screening for unmodified (WT, 531 bp) and correctly modified (ΔF508, 531 bp) sequences, and control dgDNA PCR (gDNA control, 312 bp). Representative clonal screening, shown by 1% agarose gels, suggested modified (green) and modified ΔF508-containing (red) CA1 clones generated from the 1ssT population. Negative control-untransfected CA1 cells and positive control-ΔF508-expressing CFBE cells. (**c**) Efficiency of the gene editing approach. 1ssT/+ and 1ssN/+ indicate 1ss populations S phase or G2/M phase synchronised, respectively, and transiently enriched. The identified ΔF508 clone was confirmed to be not pure, thus it was used to generate a second round of subclones, from which 75% were confirmed pure ΔF508 clones. (**d**) Sanger sequencing analysis confirmed unmodified WT (CTT highlighted in grey) and potential homozygous (orange dotted line) ΔF508 clones. (**e**,**f**) Representative WT and ΔF508 edited CA1 clones were stained for TRA-1-60 and SSEA4 pluripotency markers analysed by flow cytometry (**e**) and immunofluorescence detected by confocal microscopy (**f**), n = 4–8 biological replicates per clone. Gates were set up at 0.1% threshold for the flow cytometry analysis based on unstained and single-stained cultures (**e**) and DAPI staining was used for immunofluorescence analyses (**f**). Images were acquired with a 40x oil objective. Scale bars, 100 µm.

When dissociating and reseeding the 1ss population, it is important to achieve a density that enables single cells to grow independently without compromising cell viability [52]. These cultures were only expanded until independent colonies or clones were observed under the dissection microscope, limiting the expansion of the clones to a minimum (Figure 3a). The single-cell dissociation and reseeding process could be performed an additional time (referred to as 2ss), to adjust the initial seeding density or to further increase the chance of isolating pure clones. Once growing colonies were observed under the microscope within the S phase (1ssT) and G2/M phase (1ssN) synchronised populations, independent clones (100–200 µm diameter) were manually isolated as colonies or pieces of colonies, rather than single cells, into different wells of a 24-well plate (Figure 3a). As soon as the clones were again visible within the individual wells that survived isolation, a small portion of each colony(s) for every clone was manually collected and used to extract direct genomic DNA (gDNA) to use for early ASPCR clonal screening (Figure 3a). The ASPCR clonal screening included three PCR reactions. One PCR reaction amplifying outside of the target site was used as a control for dgDNA extraction for each clone. Two PCR reactions, relying on selective primers amplifying for either the WT or ΔF508-containing alleles, were used to detect the absence or presence of the integrated ΔF508 mutation (Figure 3b). This ASPCR screening identified a correctly modified clone containing only ΔF508 alleles (1/23 screened clones) from the S phase synchronised and transiently selected CA1 cell population with ~3% efficiency 10 days after isolation (Figure 3c). Sanger sequencing analysis confirmed the integration of the ΔF508 mutation in this clone. As part of quality control, several other clones were also analysed by Sanger sequencing, confirming the WT sequence in unmodified clones (Figure 3d) and demonstrating that most TALEN-induced indels corresponded to deletions (Figure S3a) ranging between 1–50 bp, with more than 50% of these corresponding to 1–6 bp deletions (Figure S3b). These small modifications were mostly identified within the TALEN spacer sequence, most frequently at position 5 and 6 with respect to the left TALEN monomer (Figure S3c). The expression of TRA-1-60 and SSEA4 pluripotency markers was observed in the WT and ΔF508 clones, confirming that the gene editing process did not compromise the pluripotency marker expression of these cells (Figure 3e,f).

### 2.4. CRISPR-Cas9 Efficiently Modified the CFTR Locus in Patient-Derived iPSC Lines

**Figure 4:**
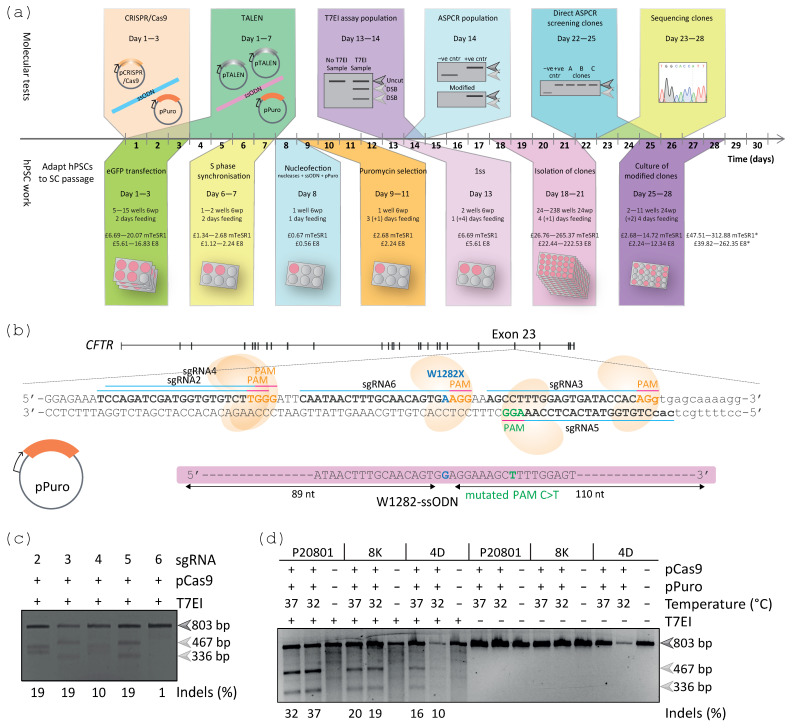
CRISPR-Cas9 showed activity at the target site in the three W1282X iPSC lines. (**a**) Stepwise timeline of the gene editing approach to introduce or correct specific mutations in the *CFTR* gene of hPSCs. * Costs of the cell culture media needed to achieve correctly modified hPSCs. (**b**) Gene editing strategy to correct the W1282X mutation (G>A, blue) into the P20801, 8K, and 4D iPSCs using CRISPR-Cas9, a ssODN harbouring the correct W1282 sequence and a pPuro. Several candidate sgRNAs (underlined) were identified complementary to the sense and the antisense strand (green and orange, respectively) followed by the respective PAM sequences. Specific CRISPR-Cas9 plasmids were generated for each sgRNA (except sgRNA1 that could not be tested due to unsuccessful cloning). (**c**) The candidate sgRNAs resulted in different Cas9 activities (467 bp and 336 bp, light grey arrows) in PlatE cells, assessed by the T7EI assay. (**d**) P20801, 8K, and 4D iPSC populations (3 × 10^6^ cells) were nucleofected with 5 µg sgRNA5-Cas9 (pCas9) and 2 µg pPuro, seeded in medium containing 5 µM Y-27632, incubated at 37 °C or 32 °C, and subjected to transient selection 1 day after nucleofection (0.7 µg/mL for 1 day and 1 µg/mL for 2 days). Only half of the amount of the amplicon could be used for the 4D 32 °C sample. The percentage of indels assessed by the T7EI assay, analysed by 3% agarose gel, indicated successful Cas9 activity in all three tested iPSC lines (467 bp and 336 bp, light grey arrows).

Using the method developed for gene editing CA1 hESCs (Figure 4a), we next aimed to use the CRISPR-Cas9 system to correct W1282X, a rare nonsense mutation present in three different iPSCs lines derived from CF patients, 8K [14], 4D [14], and P20801 [53]. For this gene editing strategy, several single-guide RNAs (sgRNAs) were identified to target a region in close proximity to the W1282X mutation within exon 23 of *CFTR* (Figure 4b). A ssODN was designed harbouring the WT sequence (W1282-ssODN) (Figure 4b), and also containing a silent mutation within the protospacer adjacent motif (PAM) to reduce the chances of repeated targeting within the corrected cells [54,55]. An independent plasmid containing puromycin resistance was also included (Figure 4b).

Firstly, the different sgRNAs were tested in the immortalised PlatE cell line, from which sgRNA5 was chosen for transfection of the iPSCs, based on the high activity observed in the PlatE cells (Figure 4c) and the close proximity to the W1282X mutation. An efficient transfection of an eGFP encoding plasmid was achieved for the three 8K, 4D and P20801 iPSC lines (Figure S4), using the B-016 nucleofection program and hSCS1, under normothermia and mild hypothermia. These iPSC lines [14] were adapted to single-cell passaging prior to this study. 8K, 4D, and P20801 iPSC lines were nucleofected with a sgRNA5-Cas9 encoding plasmid (pCas9) and pPuro, followed by incubation under normothermia (37 °C) or mild hypothermia (32 °C), and subjected to transient puromycin selection. Cas9 activity was confirmed at the target site in all three iPSC lines and conditions (37 °C and 32 °C) by the T7EI assay shown by 10–37% alleles containing indels (Figure 4d).

### 2.5. Identification of Clones Containing the Corrected W1282X Mutation in 8K, 4D, and P20801 iPSC Lines

**Figure 5:**
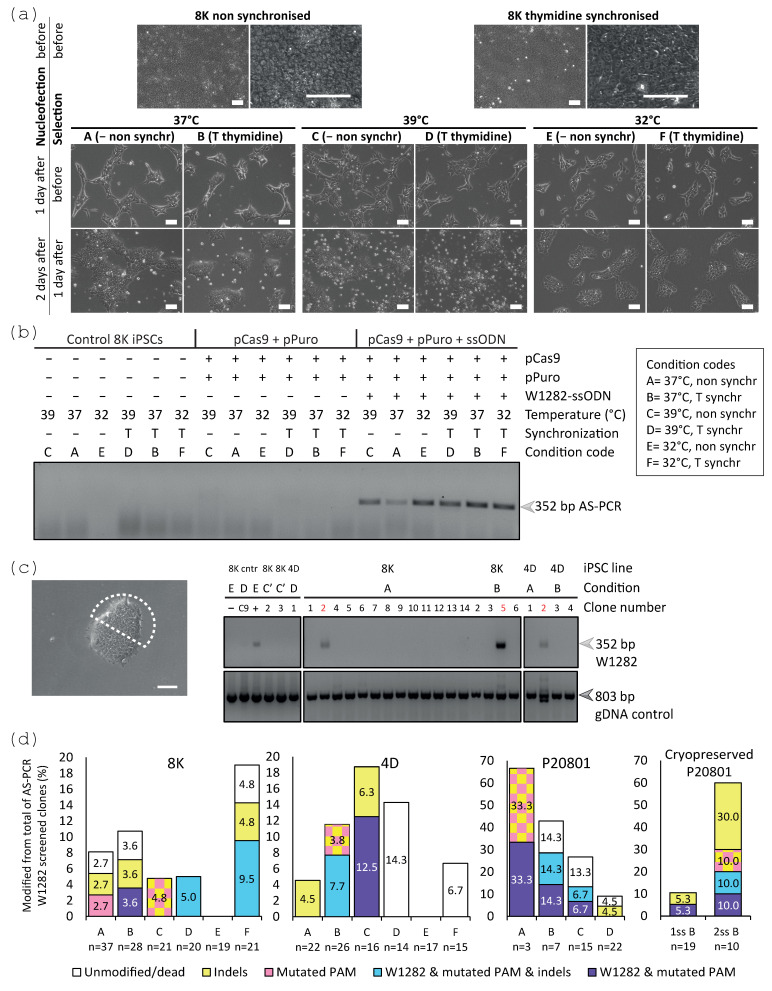
S phase synchronisation and transient selection enabled identification of corrected clones for the three iPSC lines. (**a**) Phase-contrast microscopy images of the non- and S phase synchronised 8K iPSC populations (see Figure S5 for 4D and P20801 iPSC populations) (3 × 10^6^ cells) nucleofected with 5 µg pCas9, 2 µg pPuro, and 3.5 µL W1282-ssODN, seeded in medium containing 10 µM Y-27632, and incubated at 37 °C, 39 °C, and 32 °C. Puromycin enrichment was performed 1 day after nucleofection (0.7 µg/mL for 1 day and 1 µg/mL for 1 day). Scale bars, 100 µm. (**b**) These populations (8K iPSCs as representative) were assessed for the correction of W1282X and introduced mutated PAM by ASPCR, which indicated correction under all conditions, analysed by 1.5% agarose gel (352 bp arrow). (**c**) After clonal isolation and dgDNA extraction (indicated by white shape; scale bar, 100 μm), rapid ASPCR screening identified correctly modified clones for all three iPSC lines (red). Representative screening (8K and 4D iPSC clones), shown by 1.5% agarose gels, included a PCR reaction to identify correctly modified clones (352 bp W1282, light grey arrow), and a dgDNA control PCR (803 bp gDNA control, dark grey arrow). (**d**) Sanger sequencing analysis (manual and TIDE analysis summarised in this graph can be found in Table S1) of the clones identified by ASPCR confirmed that, among others for specific iPSC lines, W1282X corrected clones were only found in all of the tested iPSC lines under condition B, including the cryopreserved P20801 iPSCs.

The three iPSC lines were synchronised in S phase prior to nucleofection with pCas9, W1282-ssODN, and pPuro. Based on a previous study reporting that hyperthermia could improve the CRISPR-Cas9-mediated gene editing efficiency [46], this condition (39 °C) was assessed after nucleofection, as well as normothermia and mild hypothermia. The iPSC populations showed a similar cell culture density under different temperatures and cell cycle synchronisation conditions, with a slightly higher density under normothermia (Figure 5a and Figure S5). P20801 iPSCs showed the lowest cell culture density after nucleofection reflecting variable susceptibility to manipulation between different iPSC lines (Figure S5). An ASPCR was optimised to identify corrected W1282X alleles and/or mutated PAM sequences, which detected correctly modified cells under all of the tested temperature and synchronisation conditions (Figure 5b). The 8K and 4D iPSC populations were dissociated and re-seeded as single cells to achieve cell culture densities that facilitate the manual isolation of independent pure clones. The nucleofected P20801 iPSC mixed populations were cryopreserved, after nucleofection (referred to as 1ss) or after transient selection prior to clonal isolation and screening (referred to as 2ss), without decreasing the gene editing efficiency.

Once the clones were isolated and colonies within each clone were identified, direct ASPCR screening was performed (Figure 5c), which included a PCR reaction to identify correctly modified clones (heterozygous and homozygous corrected) and a dgDNA control PCR. ASPCR identified correctly modified clones for each of the iPSC lines (Figure 5c) and the cryopreserved 1ss P20801B and 2ss P20801B iPSCs (Figure 5d). The identified clones were further analysed by Sanger sequencing and the chromatograms of all iPSC clones were analysed manually and using TIDE (Figure S6). Sanger sequencing confirmed clones containing the corrected W1282X and/or mutated PAM sequence for each iPSC line (Figure 5 and Table S1), although a universally favourable condition for generating correctly modified clones in the absence of additional indels was not identified. As shown by sequencing analysis, some clones identified by the ASPCR screening contained additional indels, mostly deletions (Figure S7a). The most frequently found deletions ranged from 10 to 50 bp (Figure S7b) centred around the PAM sequence (Figure S7c).

**Figure 6:**
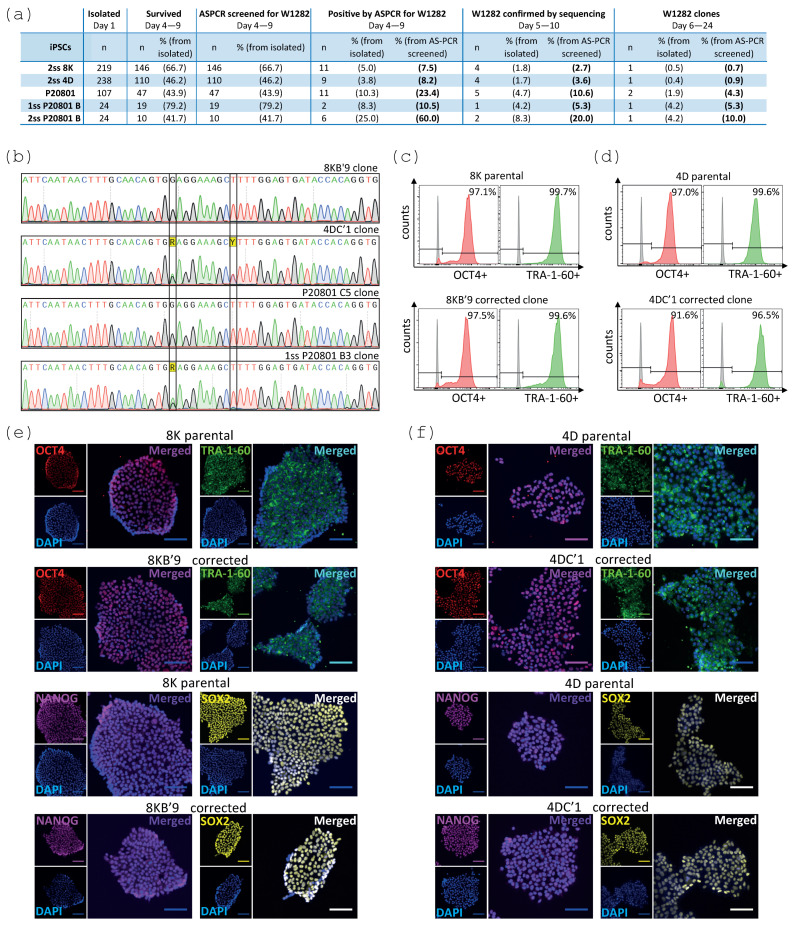
Corrected W1282X clones were confirmed by Sanger sequencing for each iPSC line and the pluripotency expression pattern was similar to the parental iPSC lines. (**a**) Efficiency of the gene editing approach for the correction of W1282X and the PAM mutation using Cas9 in iPSCs; all iPSC populations were transiently enriched. (**b**) Correction of W1282X (enclosed in first black box) and mutation of the PAM sequence (second black box) were confirmed in the 8K, 4D, and P20801 iPSC clones by Sanger sequencing. (**c**,**d**) The expression of the OCT4 (red) and TRA-1-60 (green) pluripotency markers in the corrected W1282 8KB’9 (**c**) and 4DC’1 clones (**d**), respectively, was similar to the parental iPSC lines, as determined by flow cytometry. Histogram thresholds were set up at 0.1% based on unfixed−unstained and fixed−stained with secondary antibody cell samples (represented by grey pick), n = 1 biological replicate per clone. (**e**,**f**) Immunofluorescence analyses confirmed the expression of OCT4 (red), TRA-1-60 (green), NANOG (purple), and SOX2 (yellow) pluripotency markers of the corrected 8KB’9 (**e**) and 4DC’1 clones (**f**), respectively, similar to the parental iPSC lines, assessed by confocal microscopy. DAPI staining was used for all samples. Images were acquired with a 10x objective. Scale bars, 100 µm.

The gene editing efficiency for the correction of W1282X was between 2.7%–20% of the ASPCR screened iPSC clones (Figure 6a). Some of the clones contained additional indels, making these clones no longer isogenic to the parental iPSC line; therefore, after excluding these clones, the adjusted efficiency of this gene editing strategy was 0.7–10% (Figure 6a). For the 8K iPSC line, a homozygous corrected-mutated PAM clone (1/146 screened clones, 0.7% efficiency) was confirmed by sequencing (Figure 6b). For the 4D iPSC line, a heterozygous corrected-mutated PAM clone (1/110 screened clones, 0.9% efficiency) was confirmed (Figure 6b). For the P20801 iPSCs, a homozygous corrected-mutated PAM (1/47 screened clones, 2.1% efficiency) was confirmed, as well as a heterozygous corrected clone homozygous for the mutation of the PAM sequence (1/47 screened clones) (Figure 6b). Heterozygous and homozygous corrected-mutated PAM clones (1/19 and 1/10 screened clones, 5.3–10% efficiency) were also confirmed within the cryopreserved P20801 iPSCs (Figure 6b). Importantly, T7EI assay results, Sanger sequencing, and TIDE analysis of the potential predicted off-target sites did not detect any indels introduced in the 8K iPSC populations nor the analysed representative 4D iPSC clone (Figure S8, Tables S2–S4). Finally, representative 8K and 4D corrected clones showed a similar expression of OCT4, TRA-1-60 (Figure 6c,d), NANOG, and SOX2 (Figure 6e,f) pluripotency markers compared with the respective parental iPSC lines.

## 3. Discussion

More robust, efficient, and transferable methods for gene editing hPSCs will facilitate further investigation of monogenic diseases. For multiorgan disorders, such as CF, they would also provide the additional advantage of generating in vitro systems that represent every affected organ [12,56] using the same initial sample. Introducing CF mutations of interest into hESCs would improve basic research and drug testing systems by providing isogenic controls to well-established hESC lines and would overcome the shortage of patient material for the study of rare mutations. The genetic correction of CF mutations in patient-derived iPSCs would additionally improve the development of personalised treatments for CF.

Here we demonstrate that gene editing can be performed in hESCs and iPSCs using TALENs and CRISPR-Cas9 in order to introduce or correct CF mutations in a relatively short time. Additionally, the reproducibility of this method was demonstrated by the successful introduction of precise *CFTR* modifications in four different hPSC lines.

**Figure 7:**
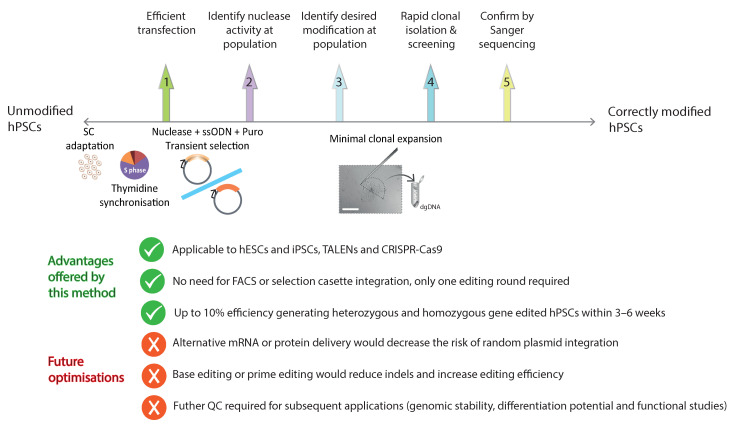
Key steps and checkpoints to generate genetically modified hPSCs in the minimum time with the maximum efficiency, including advantages offered by this method and future optimisations.

Efficient gene editing of hPSCs can be achieved by following several key steps (Figure 7). Step one is to achieve efficient transfection of the cell line of interest by optimising parameters such as transfection methods, transfection buffers, amount of plasmid (or other preferred editing material), and number of cells used for transfection. For hPSCs specifically, physical methods such as nucleofection achieve a high efficiency for plasmid transfection [22,24,26,28,29]. In particular, the B-016 nucleofector program in combination with Ingenio^®^ electroporation and hSCS1 solutions were efficient at transfecting CA1 hESCs and iPSCs, respectively.

Step two is to achieve and detect the nuclease activity at the population level. The gene editing strategy should be specifically designed for each particular need, given that each target-specific nuclease, base editor, or prime editor would impose different requirements. The advantages, applications, and remaining challenges for these newer technologies are extensively reviewed elsewhere [57,58]. In brief, base editing strategies enable A·T to G·C and C·G to T·A changes without the need to introduce DSBs, and prime editing strategies expand these capabilities to enable most single base pair changes, in addition to insertions of up to 44 bp and deletions of up to 80 bp, without the need for DSBs [9]. However, there are still applications that rely on HDR pathways upon the activity of target-specific nucleases or nickases. Examples of these would be the introduction of large modifications to generate reporter lines [59], embryos [60], or animal models, using a large ssODN or dsDNA donor template, as well as to target genes in tightly packed DNA or heterochromatin regions, where TALENs outperform Cas9 [61]. Applications that require wide target ranges or non-selective targeting, would specifically benefit from TALEN-mediated approaches, given that TALENs can be designed to target almost any given DNA sequence [62]. Among the numerous editing approaches currently available, this study focused specifically on further enhancing HDR-based gene editing outcomes in hPSCs using TALEN and CRISPR-Cas9 systems.

For approaches relying on the HDR pathway to generate knock-ins, the success of the gene editing outcome will be highly determined by the efficiency of DSBs introduced at the target site and the correction of these DSBs with the provided template. In the case of TALENs, we observed that hypothermia and mild hypothermia improved TALEN activity, which aligned with previous studies [2,44,45], where cold-shock upon transfection increased the steady-state amount of nucleases due to the accumulation of the nuclease proteins [43]. It was also confirmed that, if needed for TALEN activity, transient temperature adjustments can be made in hPSCs, without compromising the pluripotency marker expression. However, when using CRISPR-Cas9, in contrast with previous studies [46] and unlike TALENs, temperature manipulations upon nucleofection did not affect the outcome of the gene editing in the tested iPSC lines, both in terms of DSBs introduced and DSB repair with the provided ssODN.

An enrichment for transfected cells was performed to increase the chances of finding correctly modified clones by selecting against untransfected cells. This advantage can be applied by engineering the nuclease and/or the DNA template plasmid to contain an antibiotic resistance cassette, or by simply adding an additional plasmid containing antibiotic resistance in the transfection mixture. As shown here, transient selection of 2–3 days was sufficient to facilitate the detection of indels and the correct integration of the desired mutation into the hPSC’s genome, enabling the generation of hPSC clones in a single round of transfection without integrating and excising selection cassettes [27].

Step three is to optimise a robust, sensitive, and selective screening assay to identify correctly modified cells at the population stage. ASPCR offers a reliable assay to discriminate between small genetic changes of two or more base pairs, although it might be more challenging for the identification of one single base pair changes, often requiring silent modifications introduced via ssODN [63].

These three first steps, achieving an efficient transfection, detecting nuclease activity, and the desired modification at the population levels, aim to ensure that clonal isolation is performed only after maximum ssODN integration is identified within the cell populations. Particularly, a sensitive, selective, and rapid screening assay would not only reduce the time needed for clonal screening, but also help identifying unsuccessful experiments at the early stages, thus avoiding a time-consuming and high-cost clonal work that will likely fail.

Step four is to perform an early manual isolation of the clones and rapid clonal screening to ensure that the time, thus the budget, needed to gene edit hPSCs is minimised. A strength of the method described here is that it avoids processes that significantly extend the cell culture time, such as single-cell isolation into 96-well plates by limiting dilution, or FACS prior to screening, and it avoids extensive clonal expansion before screening. Instead, populations containing correctly modified cells can be dissociated and reseeded as single cells into an individual well where independent clones can form and grow within shorter culture periods than if single cells were isolated into independent wells [43]. Additionally, collecting dgDNA from growing clones within minutes to perform early clonal ASPCR screenings significantly reduced the high-workload required for screening/culturing 24–238 wells to as little as ~4 days. This made it possible to discard unmodified clones at a very early stage, resulting in the maintenance of only 2–11 wells of potentially correctly modified clones on subsequent days. The limited cell culture time, early manual clonal isolation, and rapid clonal screening, not only reduced workload but also costs (£40-313/hPSC line), enabling the early isolation and profiling of targeted hPSC clones in as little as 3 weeks.

Step five is to confirm the modifications by Sanger sequencing analysis. Having an optimised sensitive and robust clonal screening method also minimises the number of clones subjected to sequencing analysis, further decreasing the time and budget needed to gene edit hPSC lines. Here, the ASPCR screening ruled out ~91% of the screened hESC clones in a single step for the ΔF508 mutation and 40–93% of the screened iPSC clones for W1282X mutation. Performing clonal screening shortly after isolation resulted in only 4–9 days of culturing negative clones, while still helping to identify at least one correctly modified clone for each of the tested hESC and iPSC lines. The screening for W1282X resulted in more false positive clones detected than for the ΔF508 mutation, which could have been due to the selective primers used for the W1282X screening being designed to bind to the modified PAM sequence as well as W1282X, instead of the W1282X mutation only. Sequencing analysis of the additional clones performed as part of the quality control process showed that most indels corresponded to deletions, both in TALEN-mediated and CRISPR-Cas9 mediated gene edited hPSCs. Aligned with previous studies [64], most observed TALEN-mediated indels were smaller than 6 base pairs and were found within the spacer region. In the case of CRISPR-Cas9 mediated indels, most indels ranged from 10 to 50 base pairs and were identified in close proximity to the PAM sequence region, highlighting the importance of designing sgRNAs in close proximity to the mutation or change of interest for successful targeting. Interestingly, it has been shown that the ‘distance effect’ can be predicted and utilised to control zygosity or tailor mutation incorporation by HDR [63].

As summarised below (Table 1), previous methods [6,28,29] can be performed in a similar time to the method proposed in this study. However, in addition to the requirement for FACS, they also reported lower efficiency than that achieved by this study [6,28,29]. Additionally, the methods described by Yang et al. [28] and Yusa [22] only achieved heterozygous clones, thus requiring a second round of gene editing to obtain homozygous clones. Other methods [25] still need to confirm the correct modification without additional small changes at the target site by sequencing, which may increase the time required and decrease the overall efficiency [25]. Similarly, the time required to perform gene editing of iPSCs was not specified by Firth et al. [24] nor by Suzuki et al. [26] but given that it included two rounds of colony isolation and screening [24] or six cycles of enrichment [26], respectively, it would be expected that they took longer than the gene editing method described in this study. A single silent blocking mutation at PAM is generally accepted, but in cases where scarless gene modification is preferred, two-step gene editing can be applied in order to correct back the introduced mutations in PAM, as previously described [27,65].

The editing efficiency achieved by the method described in this study could be further increased by improving each of the intermediate key steps required for gene editing (Figure 7): delivery, nuclease activity, or integration of the desired modifications. Simple strategies, such as adding 1–2% DMSO to the cultures at the time of transfection [66], appeared to benefit the efficiency of NHEJ and HR upon nucleofection [48,67]. Alternatively, although this is not always the case with mRNA [68], the delivery of nucleases into the cells in the form of mRNA or protein might further improve the editing efficiency [69,70], while overcoming the risk of random plasmid integration, which was observed in some cases in this study (Figure S9). Using different TALEN architectures [2], such as GoldyTALEN [71] or SunnyTALEN [72], could further enhance the final editing efficiency. Other alternatives could be to use a synthetically modified sgRNA and donor DNA or sgRNA and donor DNA conjugated into one molecule [73]; to use the Cas9-Avidin-Biotin ssDNA (CAB) system, which reported an increase in the knock-in efficiency by 3–5 fold in mouse zygotes or cells, respectively [74]; or the Cas9-streptavidin-biotin approach to localize dsDNA repair templates to targets sites, which reported a knock-in efficiency of large fragments up to 95% in mouse blastocysts [60].

A final parameter to mention from this study is that all of the hPSC lines used here were previously adapted to single-cell passaging, which may have enhanced the gene editing efficiency by increasing their transfection efficiency compared with those cells passaged as clumps using EDTA. Synchronisation of the cell cycle in S or G2/M phases using thymidine [51] and nocodazole [48] might be advantageous for some hESC/iPSC lines, but not others, as observed in this study; therefore, these conditions can be explored as an option for individual cell lines on a case-by-case basis. Variability on the editing efficiency is also expected between different iPSC lines, as previous studies have identified this variability even when correcting/introducing the same mutation in different iPSC lines [70]. Although the approaches highlighted above have previously been extensively described in previous iPSC studies, a potential limitation of this study is the lack of further characterisation of the generated iPSC lines. Besides confirming genetic modifications in hPSC-derived clones, potential random integrations or mutations and off-target effects [75] of the derivative cells compared with the parental hPSC lines, additional quality controls are recommended when gene edited cells are intended for use as disease models in order to identify any potential issues that might have occurred during the process. Quality controls often include confirmation of genomic stability, differentiation into the lineage of interest, and functional studies. For applications where higher levels of safety are mandatory, alternative approaches to nuclease-mediated gene editing that impose even less risk of off-target effects such as in trans paired nicking [76] or base editing [7,8] might be preferred options.

## 4. Materials and Methods

### 4.1. Gene Editing Tools

TALENs were engineered using the Golden-Gate assembly method, as previously described [3,77]. TALEN monomer plasmids were used at a 1:1 ratio for transfection. For CRISPR-Cas9, the sgRNAs identified by the Optimized CRISPR design tool (this tool is no longer available, but similar resources can be found at (https://www.zlab.bio/resources), accessed on 12 June 2023) were annealed and cloned into pX330 vectors (a kind gift from E. Zhang; Addgene plasmid 42230), as previously described [5].

The ssODNs were obtained from Integrated DNA Technologies (Coralville, IA, USA) and reconstituted as a 100 µM stock in nuclease-free ddH2O. The pPuro, used for CA1 cells, was kindly donated by C. Lee (Rice University, Houston, TX, USA). The pPuro used for iPSCs was generated by replacing the Cas9 cassette for puromycin resistance cassette in the Addgene plasmid 42230.

### 4.2. Cell Culture

The CA1 hESC line was provided by the Nagy lab (Mount Sinai Hospital, Toronto, ON, Canada) (https://hpscreg.eu/cell-line/MSHRIe001-A, accessed on 12 June 2023). The 8K and 4D iPSC lines were generated and provided by the Centre for Commercialization of Regenerative Medicine (CCRM) in collaboration with The Cystic Fibrosis Canada-SickKids Program for Individualized CF Therapy (CFIT) (https://lab.research.sickkids.ca/cfit/cystic-fibrosis-patients-families-researchers/cell-resources-available/, accessed on 12 June 2023) [14]. The PCMD20801 iPSC line (referred to as P20801, https://www.seas.upenn.edu/~diamond/iPS%20P20801%20Cell%20line%20description.pdf, accessed on 12 June 2023) was generated by the University of Pennsylvania in collaboration with Emily’s Entourage [53]. Human PSCs were cultured on Matrigel^®^ matrix (#354277, Corning, New York, NY, USA) coated tissue-culture plates, CA1 hESCs in mTeSR™1 medium (#85851 and #85852, STEMCELL Technologies™, Vancouver, BC, Canada), and iPSCs in Essential 8™ medium (#A1517001, Thermo Fisher Scientific, Waltham, MA, USA), with daily media changes [34]. CA1 cells were routinely passaged by dissociation into small clumps using EDTA (Versene®-EDTA, 0.02%, #17-711E, Lonza, Basel, Switzerland) (referred to as EDTA CA1 cells) or dissociated into single cells using StemPro™ Accutase™ Cell Dissociation Reagent (#A1110501, Thermo Fisher Scientific, Waltham, MA, USA) (referred to as CA1 cells). Human iPSCs were routinely dissociated into single cells for passaging using Gentle Cell Dissociation Reagent (#07174, STEMCELL Technologies™, Vancouver, BC, Canada).

### 4.3. Cell Cycle Synchronisation

S phase cell cycle synchronisation was achieved by double-thymidine treatment, consisting of 2 mM thymidine (#T1895, Merck, Kenilworth, NJ, USA) for 14.5 h treatment, released for 9 h, followed by 2 mM thymidine treatment for additional 16 h. No release time was included between double-thymidine treatment and nucleofection. Cell cycle synchronisation at the G2/M phase was achieved using nocodazole (#M1404, Merck, Kenilworth, NJ, USA) at 11 µM final concentration for 16–16.5 h at 37 °C. Cultures were released from nocodazole treatment for 40–120 min prior to nucleofection. Non-synchronised cultures were maintained in parallel under standard conditions.

### 4.4. Nucleofection and Temperature Conditions

Nucleofection solutions were prepared with 2.5–10 µg plasmid DNA (eGFP, TALENs, CRISPR-Cas9) resuspended in 100 µL total volume of the indicated buffer. All hPSCs were dissociated into single cells and counted prior to nucleofection. Nucleofection was performed using Nucleofector™ II/2b Transfection Device (#AAB-1001, Lonza, Basel, Switzerland), Ingenio® electroporation solution (#MIR 50111, Mirus Bio, Madison, WI, USA) for CA1 cells, and human Stem Cell solution 1 (hSCS1, #VPH-5002, Lonza, Basel, Switzerland) for iPSC, and B-016 program, unless otherwise stated. Cells subjected to nucleofection were then gently resuspended in mTeSR™1 or E8 media (CA1 or iPSC, respectively) containing 5–10 µM ROCK Inhibitor (Y-27632, #72302, STEMCELL Technologies™, Vancouver, BC, Canada) [78,79] and seeded at a high density (1:1, 1:2, or 1:3 wells of 6-well plate(s)). Y-27632 was not maintained in the media for more than 24 h. Following nucleofection, human PSC populations were incubated at normothermia, mild hypothermia, hypothermia, or hyperthermia. Normothermia was defined as 37 °C; mild hypothermia was defined as 3h at 37 °C after nucleofection, followed by 2 days at 32–33 °C, followed by incubation at 37 °C until passage or analysis; hypothermia was defined as 3h at 37 °C after nucleofection, followed by 2 days at 30 °C, followed by incubation at 37 °C until passage or analysis; hyperthermia was defined as 2 days at 39 °C after nucleofection, followed by incubation at 37 °C until passage or analysis.

### 4.5. Transient Puromycin Selection

Selection was performed using puromycin (#A1113802, Thermo Fisher Scientific, Waltham, MA, USA) 1–2 days after nucleofection at a final concentration of 0.7–1 µg/mL for 2–3 days, depending on the cell density of each culture post nucleofection.

### 4.6. Clonal Isolation

Isolation of clones was done under a stereomicroscope built into a class I or class II safety cabinet. The isolation was performed manually by detaching and transferring each single colony using a 10 μL plastic tip or a Stem Cell Cutting Tool (#14601, Vitrolife, Göteborg, Sweden) into a separate well of 24-well plate and treated as clones thereafter. If colonies were big (300 × 300 μm), they were cut into several pieces to facilitate attachment.

### 4.7. Genomic DNA Extraction

Extraction was performed following protocols based on the manufacturer’s recommendations; Wizard^®^ Genomic DNA purification kit (#A1120, Promega, Madison, WI, USA) for CA1 and CFBE populations, REDExtract-N-Amp™ Tissue PCR kit (#XNAT Merck, Kenilworth, NJ, USA) for iPSC populations, Phire Tissue Direct PCR Master Mix (#F170S, Thermo Fisher Scientific, Waltham, MA, USA), following the manufacturer’s dilution protocol, was used for direct gDNA extraction from the hPSC clones. The extracted gDNA was used to perform PCR reactions with several purposes, including sequencing analysis (Table S1). Allele-specific PCR (ASPCR) was performed using primers designed to selectively amplify the unmodified or correctly modified sequences (Table S1). The results were analysed by 1–1.5% agarose gel electrophoresis.

### 4.8. T7 Endonuclease I Assay

The assay was performed using the gDNA extracted from the hPSC populations and following the NEB manufacturer’s protocol of the T7 Endonuclease I (T7EI, #M0302S, New England BioLabs, Ipswich, MA, USA). In brief, the regions containing the desired target sites were amplified using primers listed in Table S5 (312 bp or 803 bp, for CA1 cells and iPSCs, respectively), and after amplicon purification, denaturation, and reannealing, and the amplicons (100–200 ng) were subsequently digested with T7EI. Cleaved products were analysed by 2.5–3% agarose gel electrophoresis. Cleavage was quantified using the image processing package FIJI and expressed as percentage of indels [80].

### 4.9. Sanger Sequencing Analysis

Sequencing was performed using primers listed in Table S5 by Source Bioscience (Nottingham, UK) for the CA1 cells and by the Centre for Applied Genomics (TCAG Facilities, SickKids, Toronto, ON, Canada) for iPSCs. The obtained chromatograms were manually analysed for CA1 and iPSCs. Additionally, most modifications were confirmed by the Tracking of indels by Decomposition [81] (TIDE, http://shinyapps.datacurators.nl/tide/, accessed on 12 June 2023) software for the CRISPR-Cas9 mediated-modified iPSCs and off-target analysis.

### 4.10. Immunohistochemistry and Confocal Microscopy

The following pluripotency markers were assessed; TRA-1-60 in the CA1 clones, and OCT4, TRA-1-60, NANOG and SOX2 in the iPSC clones, and analysed by confocal microscopy. In brief, samples were grown and fixed on glass coverslips for the CA1 cells (or directly fixed on the 24-well cell culture plates for iPSCs) with 4% paraformaldehyde (PFA, #1.04005.1000, Merck, Kenilworth, NJ, USA) in PBS (15 min at 37 °C or for 10 min at RT for iPSCs). After three PBS washes, the samples were permeabilised with 1% Triton-X (#T8787, Merck, Kenilworth, NJ, USA) in PBS at RT for 15 min (or with 0.1% Nonidet P-40 in PBS at RT for 10 min, followed by three washes at RT for 10 min with PBS-T (PBS + 0.1% Tween® 20, #P2287 Merck, Kenilworth, NJ, USA) for iPSCs). The samples were then blocked for 1 h at RT with 5% BSA (#A1470, Merck, Kenilworth, NJ, USA) in PBS-T (or 6% normal goat serum and 0.5% BSA in PBS-T for iPSCs), incubated with the following primary antibodies; rabbit anti-human OCT-3/4 (1:200 dilution), rabbit anti-human NANOG (1:100 dilution, #Ab21603 Abcam, Cambridge, UK), rabbit anti-human SOX2 (1:200 dilution, #GTX101507, GeneTex, Irvine, CA, USA), and mouse anti-human TRA-1-60 (1:100 dilution, #41-1000, Thermo Fisher Scientific, Waltham, MA, USA) in blocking solution overnight at 4 °C and 2–4 h at RT. After three PBS-T washes, the samples were incubated with secondary antibodies (diluted in PBS at 1:400 for anti-rabbit AF555 (#A21429, Thermo Fisher Scientific, Waltham, MA, USA), at 1:500 for anti-rabbit AF488 (#A-11008, Thermo Fisher Scientific, Waltham, MA, USA), for anti-mouse AF555 (#A21422, Thermo Fisher Scientific, Waltham, MA, USA), and AF546, for anti-rat AF647 (#A21248, Thermo Fisher Scientific, Waltham, MA, USA) in blocking solution in the dark for 2 h (1 h for iPSCs). Finally, after three more washes with PBS-T and one with nuclease-free ddH2O, the samples were mounted on one drop of ProLong™ Gold Antifade Mountant with DAPI (#P36931, Thermo Fisher Scientific, Waltham, MA, USA) (or treated with DAPI at 2 µL/mL at 10 min at RT, followed by two additional washes in PBS, and stored in PBS for 4 h at 4 °C before imaging for iPSCs). The samples were left to dry overnight before confocal microscopy analysis or they were stored at −80 °C (or stored in PBS at 4 °C for iPSCs). Immunofluorescence images were acquired using the 40X and 10X objectives on a Nikon A1R confocal microscope using NIS Elements.

### 4.11. Immunostaining and Flow Cytometry

The following pluripotency markers were assessed; TRA-1-60 and SSEA4 in the CA1 clones, and OCT4 and TRA-1-60 in the iPSC clones and analysed by flow cytometry. In brief, the cells were fixed with 1 mL of 2% PFA for 15 min at 37 °C or resuspended in 1 mL ice-cold methanol (#A411-4 Thermo Fisher Scientific, Waltham, MA, USA) and incubated for 30 min to overnight at −20 °C for permeabilization and washed once with 10 volumes of PBS. The cells were centrifuged, and pellets were resuspended in 50–200 µL flow cytometry buffer (PBS 2% FBS) for incubation with the following primary antibodies for iPSC pluripotency marker expression; rabbit anti-human OCT-3/4 (1:200 dilution) and mouse anti-human TRA-1-60 (1:100 dilution), followed by the secondary antibody staining, as previously described. Conjugated antibodies were used instead for CA1 cell pluripotency marker expression, mouse anti-human TRA-1-60-AF^®^555 (1:50 dilution, #560121, BD Pharmingen™, San Diego, CA, USA), and mouse anti-human SSEA-4-PerCy™ 5.5 (1:50 dilution, #561565, BD Pharmingen™, San Diego, CA, USA) incubated in the dark for 30–120 min at 4 °C. The samples were washed to remove free antibody and DAPI (1:10–20 dilution, #D3571, Thermo Fisher Scientific, Waltham, MA, USA) was added to each sample.

Additionally, flow cytometry was also used to quantify transfection efficiency by directly collecting nucleofected populations, as well as controls, in a 500 µL flow cytometry buffer and all of the samples were stained with DAPI (1:10 dilution) for the analysis.

### 4.12. Statistical Analysis

The box plots were generated for visual purposes; however, independent statistical analyses were performed using GraphPad Prism 7. Nonparametric Mann–Whitney U test and Kruskal–Wallis tests were used to compare two or three groups, respectively, when in at least one of the groups, mean and median differed, distribution was skewed, or it contained less than 15 data points. Note that significant differences were accepted when *p* ≤ 0.05, although exact *p* values are indicated for each experiment.

## 5. Conclusions

In summary, we demonstrate here that small and transient manipulations can be incorporated in current gene editing approaches in hPSCs without compromising the viability or the pluripotency marker expression crucial for subsequent experiments on the gene edited hPSCs. Importantly, our study demonstrates that this method can be applied to both hESCs and iPSCs using TALENs or CRISPR-Cas9, achieving high editing efficiencies without using FACS or long cell culture periods. We speculate that the method described here can also be used to target different regions of the *CFTR* gene, modifier genes, or other genes in order to model other genetic diseases. This study demonstrates that heterozygous or homozygous genetic changes can be introduced to generate or correct specific mutations with only one round of clonal isolation. We also show that this method is amenable to different gene editing technologies, and we anticipate that it could be expanded to other CRISPR systems, different TALEN architectures [2], or even the newer base editing [7,8] and prime editing [9] systems. Overall, we describe a rapid, robust, and reproducible method for gene editing hPSCs that can subsequently be used for disease modelling, benchmark mutation-related function studies, or gene repair for regenerative medicine.

## Figures and Tables

**Table 1:** Comparison of current methods for gene editing hPSCs and the method described in this study. Aspects where the method described here offers an advantage are highlighted in grey. Induced pluripotent stem cells (iPSCs), human embryonic stem cells (hESCs), nucleofection (N), and electroporation (E).

Cell Type	Transfection Method	Nuclease	Integrated Selection Cassette	FACS	Number of Screened Clones	Efficiency	Time Required	References and Comments
iPSCs	N	reTALENs, CRISPR-Cas9	No	Yes	100	0.6–1.7%	3 weeks	Clones reported using re-TALENs [28]
								Only heterozygous clones
hESCs	E	TALEN	No	Yes	192	1.60%	Less than 1 month	Ding et al. [6]
iPSCs	N	CRISPR-Cas9	eGFP or dTOMATO	Yes	24	1st transfection (2.2–3.8%) + 2nd transfection (2.2–6.5%)	More than 2 weeks, not specified	Clones derived from correctly modified ‘polyclones’ previously identified by FACS [29]
hESCs	N	ZFN, TALEN	Puromycin	No	24	Not specified, but two rounds of transfection	~3 months	Only heterozygous clones [22]
iPSCs	N	CRISPR-Cas9	eGFP-puromycin	No	36	1st transfection (16.7%) + 2nd transfection (1–88.1%)	Not specified, but 2 rounds of colony isolation and screening	Firth et al. [24]
hESCs	E	ZFN, TALEN, CRISPR-Cas9	Puromycin-eGFP	No	150–412	87–96% of eGFP+ clones	20–24 days to identify integrated selection cassette	Only PCR verified modifications [25]
						2 rounds of transfection		
iPSCs	N	TALEN	No	No	30	20% after 6 enrichment cycles	Not specified, but 6 enrichment cycles from 1st observation of correction (day 9 after transfection) to isolation of clones	Each cycle consisted of cell dissociation, seeding as clumps and PCR screening [26]
hESCs, iPSCs	E	CRISPR-Cas9	No	Yes	1st isolation (96–192) + 2nd isolation (384–96)	1st transfection (2.9–9.6%) + 2nd transfection (7.7–15.4%)	~3 months	2 rounds of transfection, to remove the introduced mutations in the PAM [65]
hESCs, iPSCs	N, FuGene HD transfection	CRISPR-Cas9	Puromycin	No	1st isolation (11) or 2nd isolation (12)	1st transfection (18.2%) or 2nd transfection (41.6%)	35 days, but 2 rounds of transfection	The authors suggest performing only the 2nd clonal screening [27]
hESCs, iPSCs	N	TALEN, CRISPR-Cas9	No	No	24–238	1–10%	~3–6 weeks	The method described here

## Data Availability

Data is contained within the article or supplementary material. Additional information available on request.

## Supplementary Materials

The following supporting information can be downloaded at: https://www.mdpi.com/article/10.3390/ijms241210266/s1.
