# Supplementary material for: A Cell-Based Optimised Approach for Rapid and Efficient Gene Editing of Human Pluripotent Stem Cells
Source: Int J Mol Sci. 2023 Jun 17;24(12):10266. doi: 10.3390/ijms241210266 (PMC10299534; doi:10.3390/ijms241210266)
Supplement: Supplementary file 1 [file ijms-24-10266-s001.zip › ijms-2426630-SM.pdf]

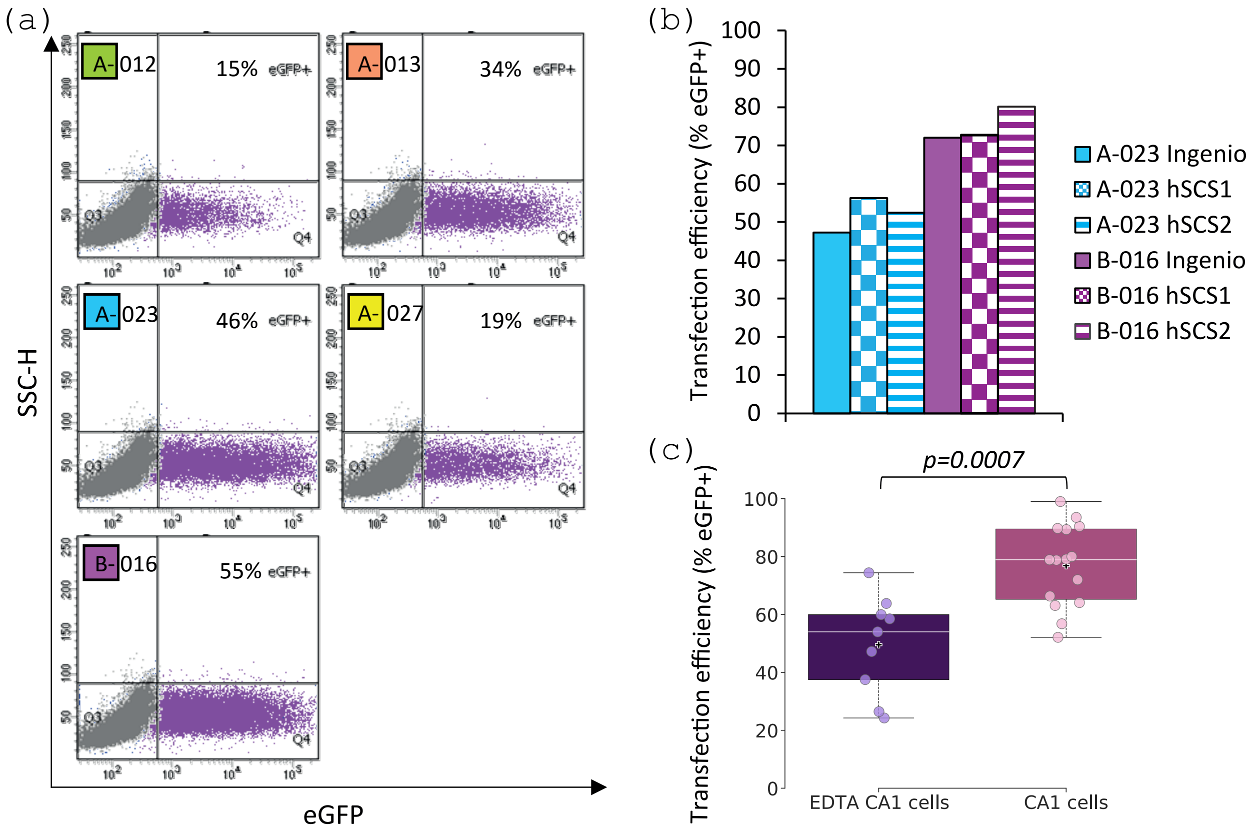

(a)
A-012
15% eGFP+
A-013
34% eGFP+
A-023
46% eGFP+
A-027
19% eGFP+
B-016
55% eGFP+
Q3
Q4
SSC-H
eGFP
(b)
Transfection efficiency (% eGFP+)
A-023 Ingenio
A-023 hSCS1
A-023 hSCS2
B-016 Ingenio
B-016 hSCS1
B-016 hSCS2
(c)
p=0.0007
Transfection efficiency (% eGFP+)
EDTA CA1 cells
CA1 cells

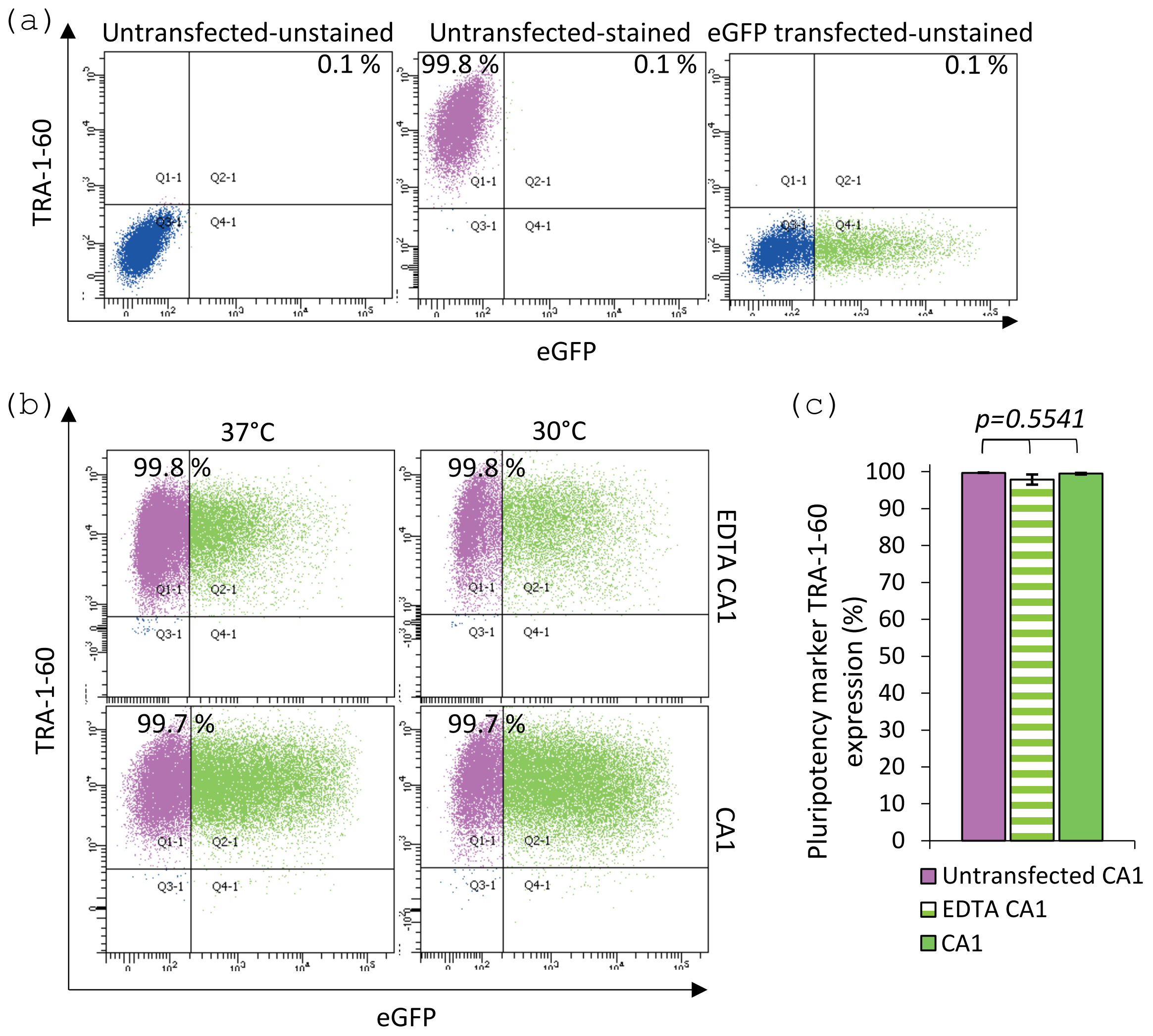

(a)
Untransfected-unstained
Untransfected-stained
eGFP transfected-unstained
0.1 %
99.8 %
0.1 %
0.1 %
TRA-1-60
eGFP
(b)
37°C
30°C
99.8 %
99.8 %
99.7 %
99.7 %
EDTA CA1
CA1
TRA-1-60
eGFP
(c)
p=0.5541
Pluripotency marker TRA-1-60 expression (%)
Untransfected CA1
EDTA CA1
CA1

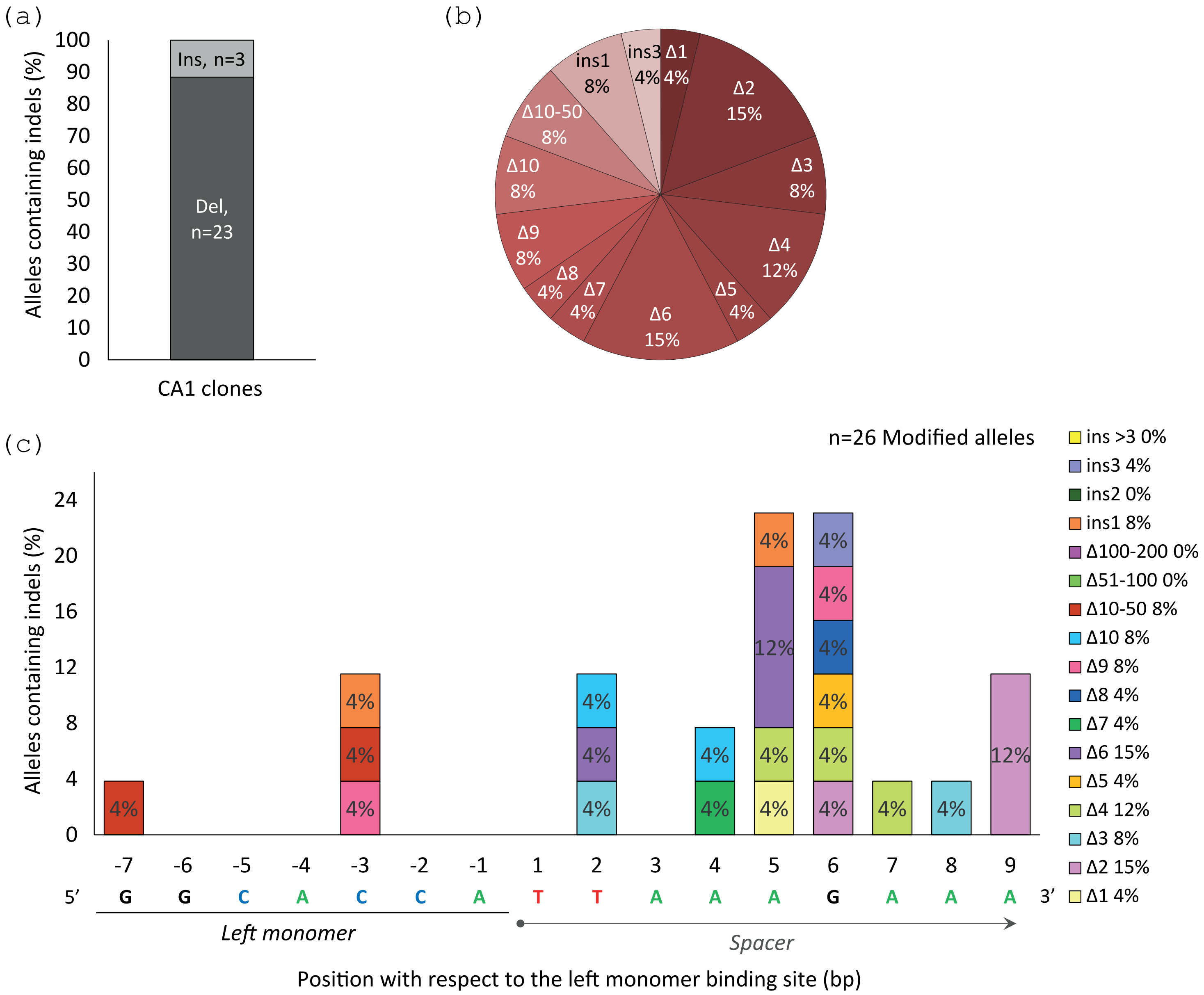

(a)
Alleles containing indels (%)
100
90
80
70
60
50
40
30
20
10
0
Ins, n=3
Del, n=23
CA1 clones
(b)
Δ1 4%
Δ2 15%
Δ3 8%
Δ4 12%
Δ5 4%
Δ6 15%
Δ7 4%
Δ8 4%
Δ9 8%
Δ10 8%
Δ10-50 8%
ins1 8%
ins3 4%
(c)
n=26 Modified alleles
Alleles containing indels (%)
24
20
16
12
8
4
0
4%
12%
-7 -6 -5 -4 -3 -2 -1 1 2 3 4 5 6 7 8 9
5' G G C A C C A T T A A A G A A A 3'
Left monomer
Spacer
Position with respect to the left monomer binding site (bp)
ins >3 0%
ins3 4%
ins2 0%
ins1 8%
Δ100-200 0%
Δ51-100 0%
Δ10-50 8%
Δ10 8%
Δ9 8%
Δ8 4%
Δ7 4%
Δ6 15%
Δ5 4%
Δ4 12%
Δ3 8%
Δ2 15%
Δ1 4%

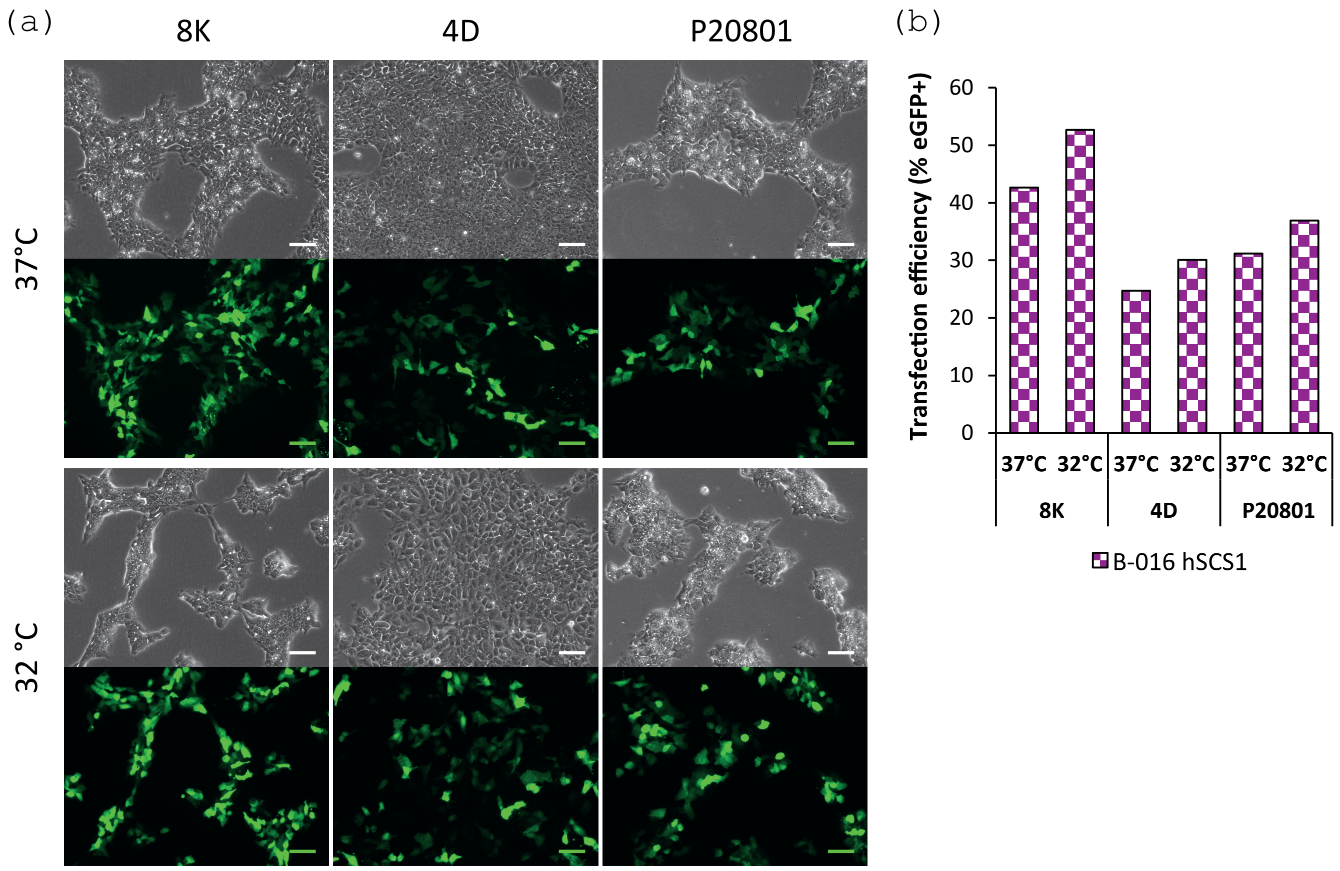

(a)
8K
4D
P20801
37°C
32 °C
(b)
Transfection efficiency (% eGFP+)
60
50
40
30
20
10
0
37°C
32°C
37°C
32°C
37°C
32°C
8K
4D
P20801
B-016 hSCS1

(a)

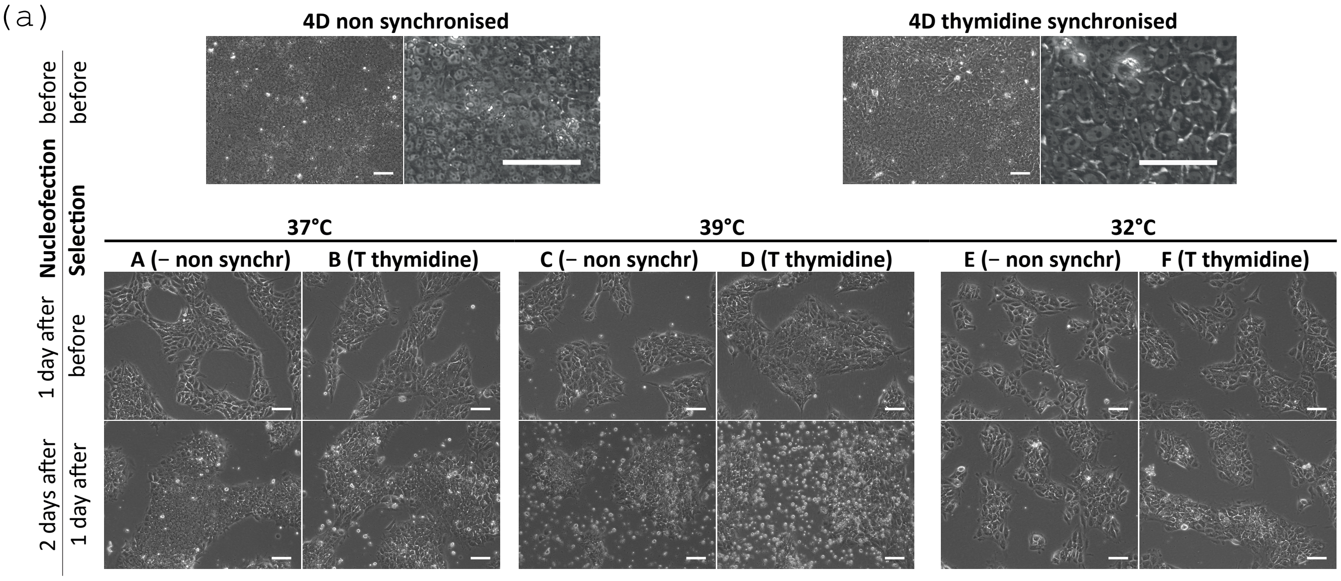
4D non synchronised
4D thymidine synchronised
Nucleofection before
Selection before
37°C
39°C
32°C
A (− non synchr)
B (T thymidine)
C (− non synchr)
D (T thymidine)
E (− non synchr)
F (T thymidine)
1 day after
before
2 days after
1 day after

(b)

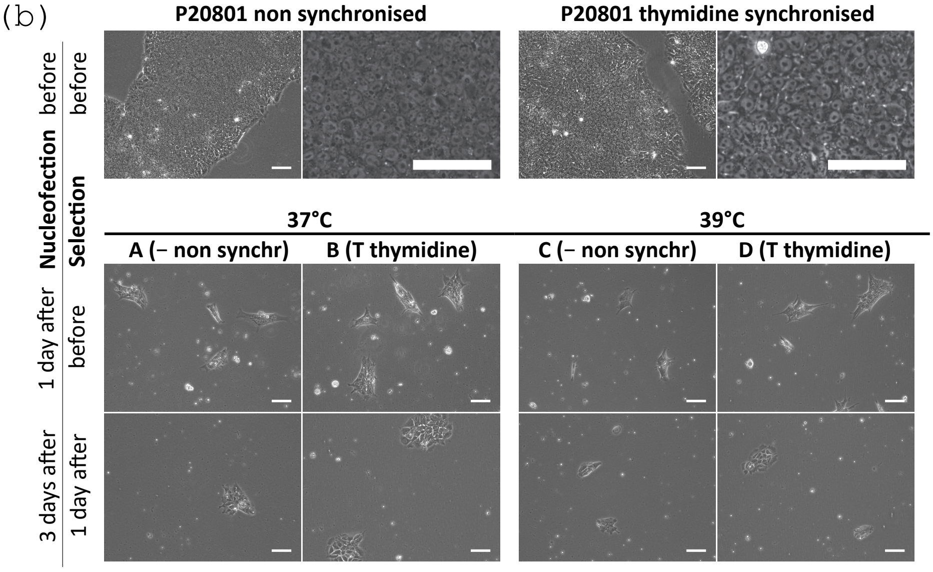
P20801 non synchronised
P20801 thymidine synchronised
Nucleofection before
Selection before
37°C
39°C
A (− non synchr)
B (T thymidine)
C (− non synchr)
D (T thymidine)
1 day after
before
3 days after
1 day after

(a)

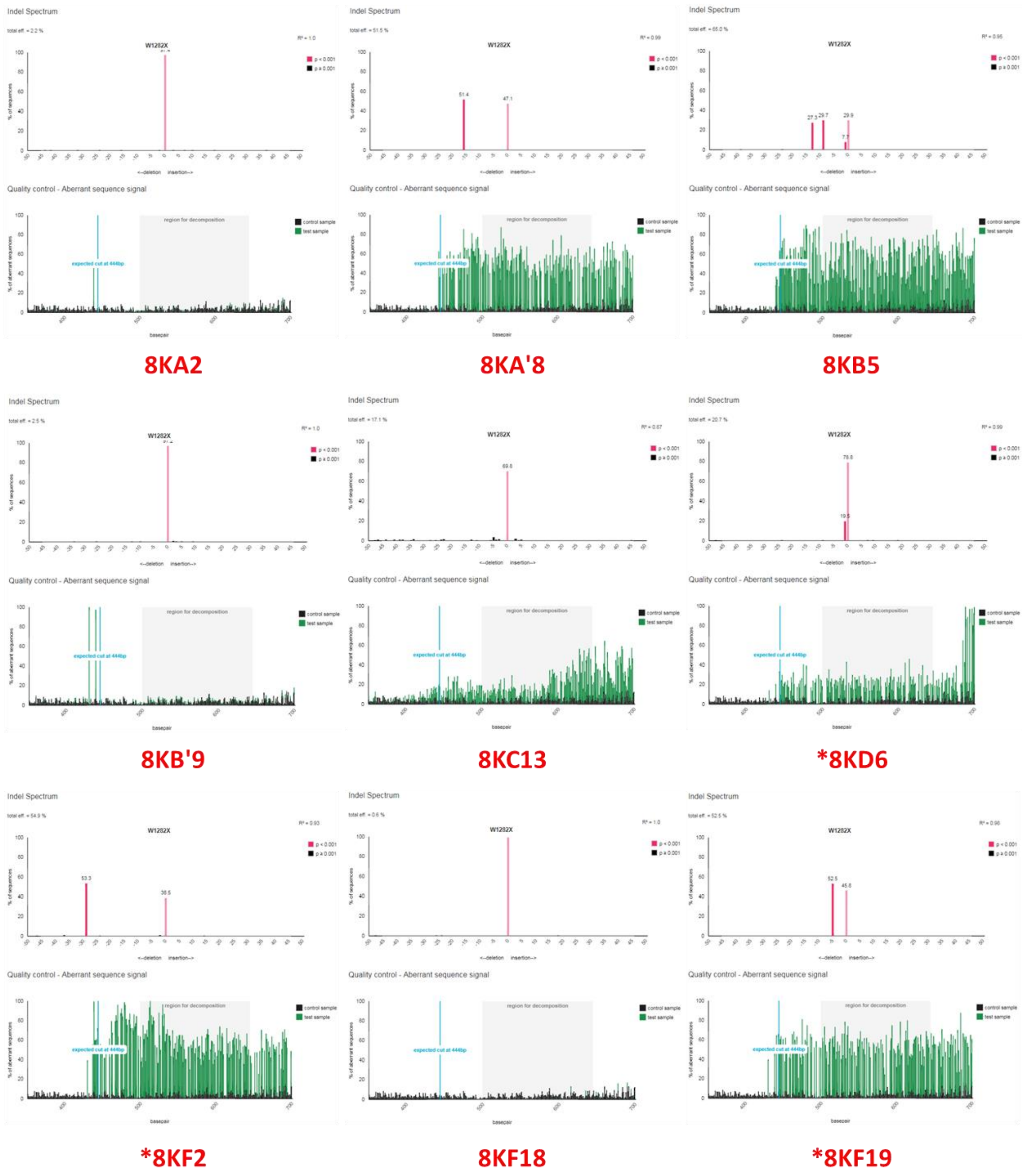

Indel Spectrum
W1282X
Quality control - Aberrant sequence signal
region for decomposition
expected cut at 444bp
control sample
test sample
8KA2
8KA'8
8KB5
8KB'9
8KC13
*8KD6
*8KF2
8KF18
*8KF19

(b)

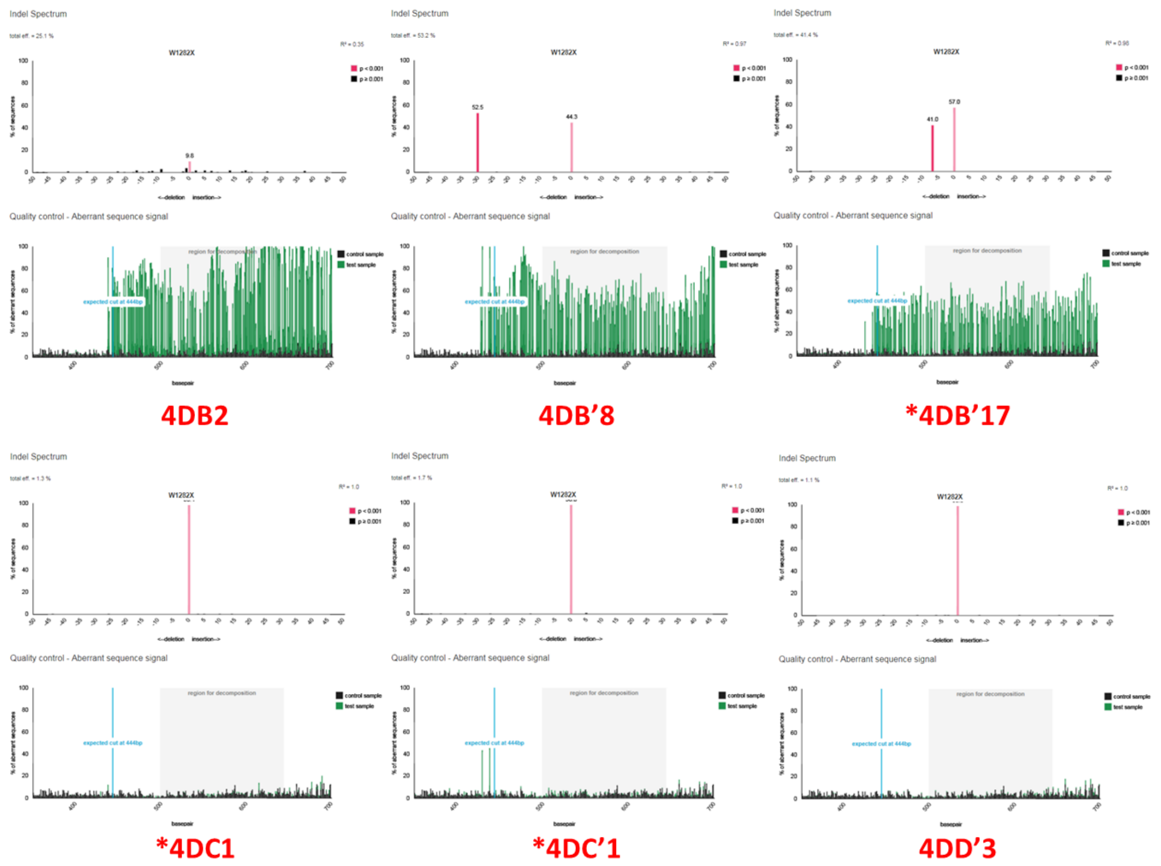
Indel Spectrum
W1282X
Quality control - Aberrant sequence signal
4DB2
4DB'8
*4DB'17
*4DC1
*4DC'1
4DD'3

(c)

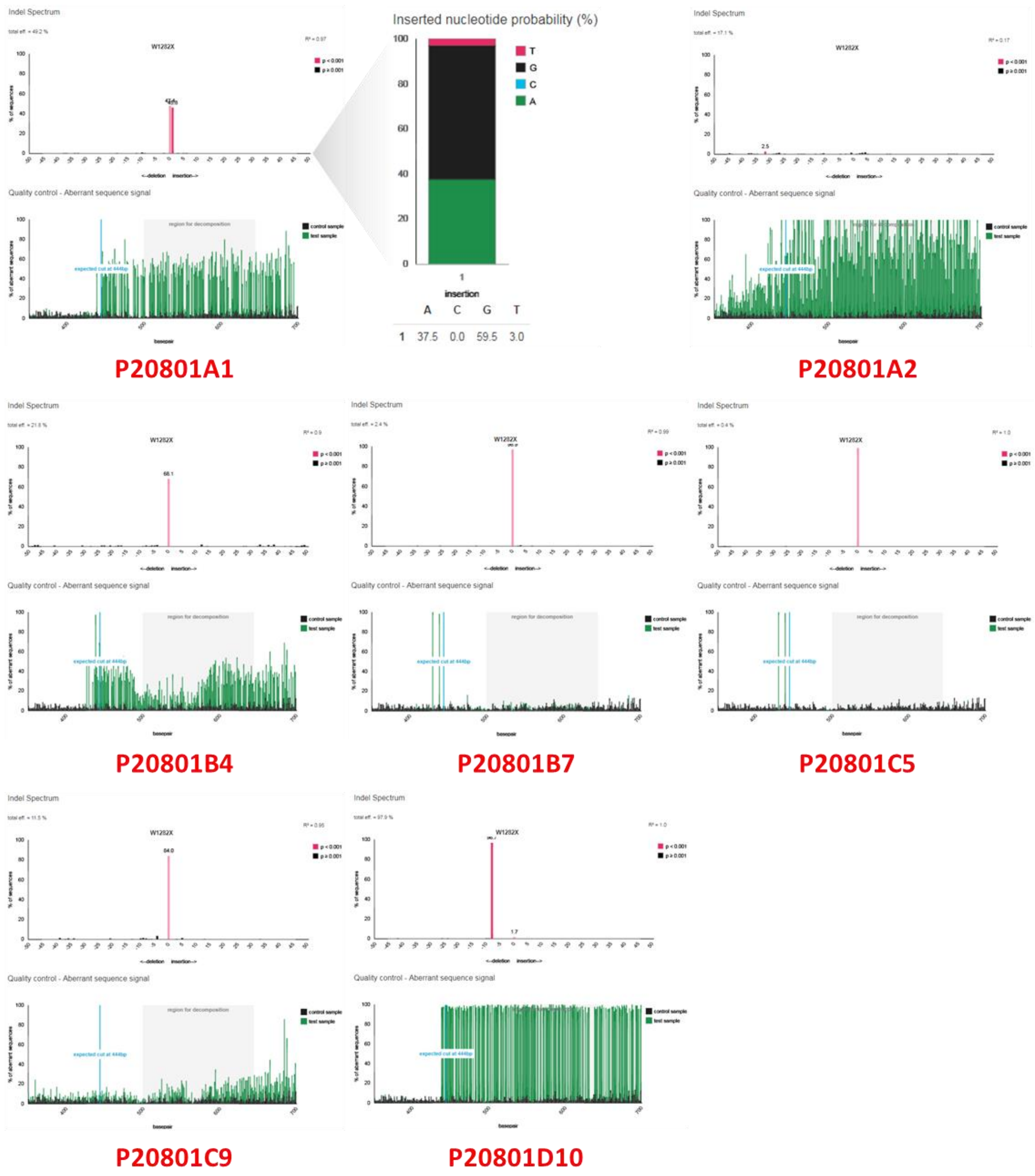
Indel Spectrum
W1282X
Quality control - Aberrant sequence signal
Inserted nucleotide probability (%)
T
G
C
A
insertion
A C G T
1 37.5 0.0 59.5 3.0
P20801A1
P20801A2
P20801B4
P20801B7
P20801C5
P20801C9
P20801D10

(d)

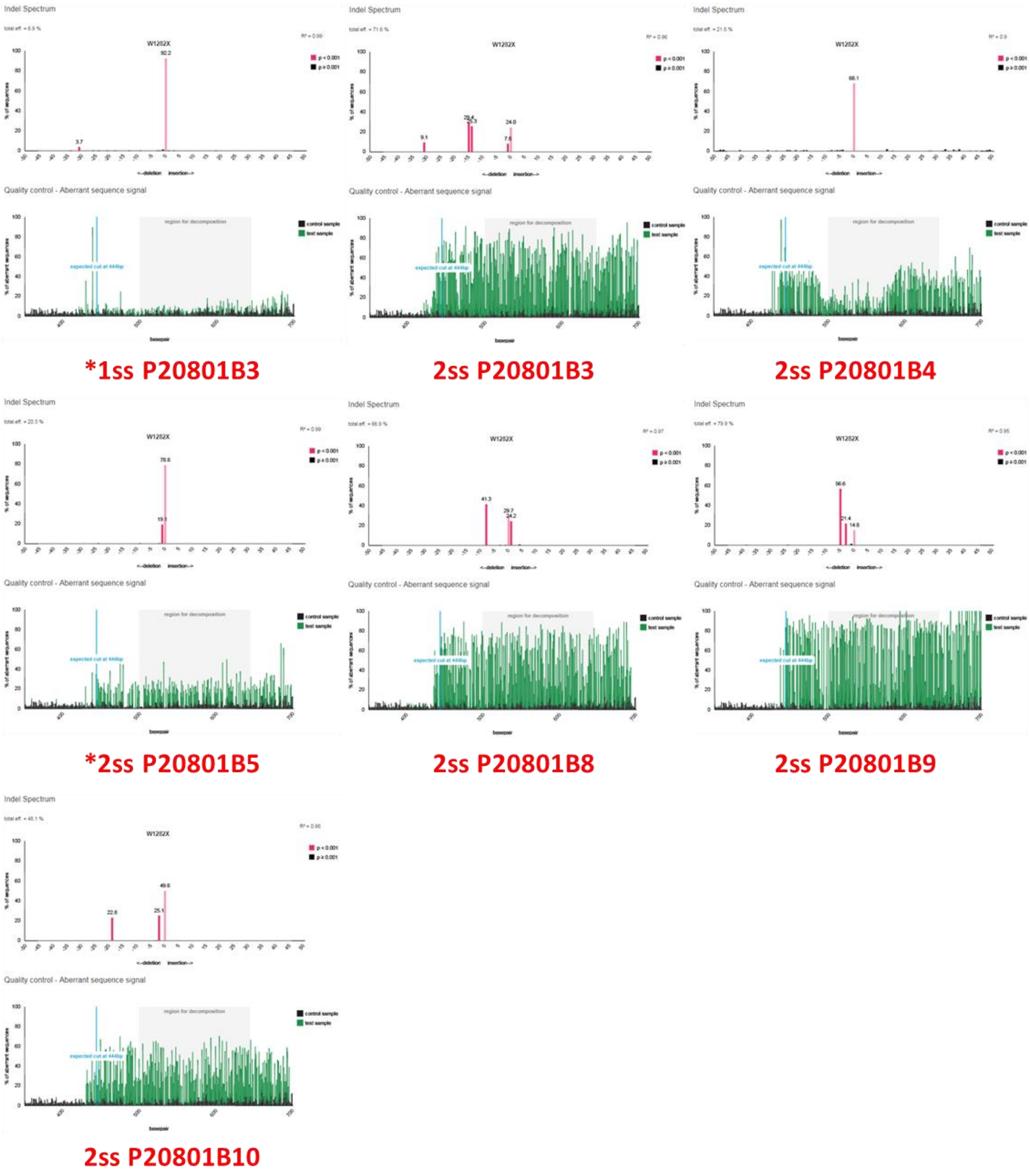
Indel Spectrum
W1202X
Quality control - Aberrant sequence signal
*1ss P20801B3
2ss P20801B3
2ss P20801B4
*2ss P20801B5
2ss P20801B8
2ss P20801B9
2ss P20801B10

(e)

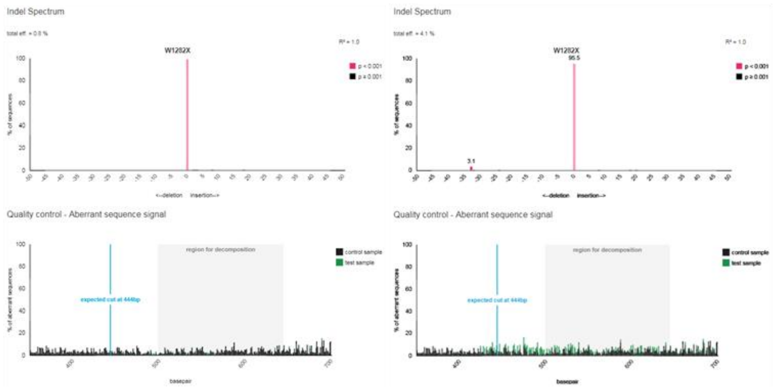

8KB'16 **4DA'9**

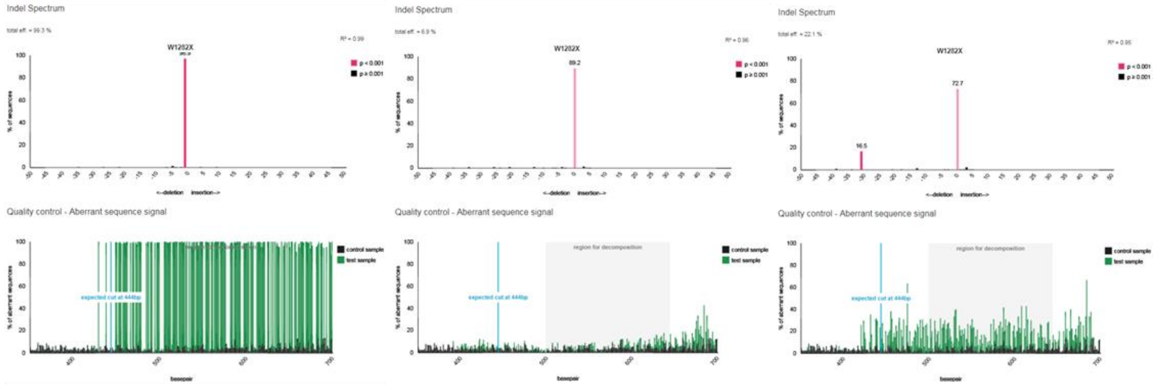

P20801C6 **P20801C8** **1ss P20801B12**

(f)

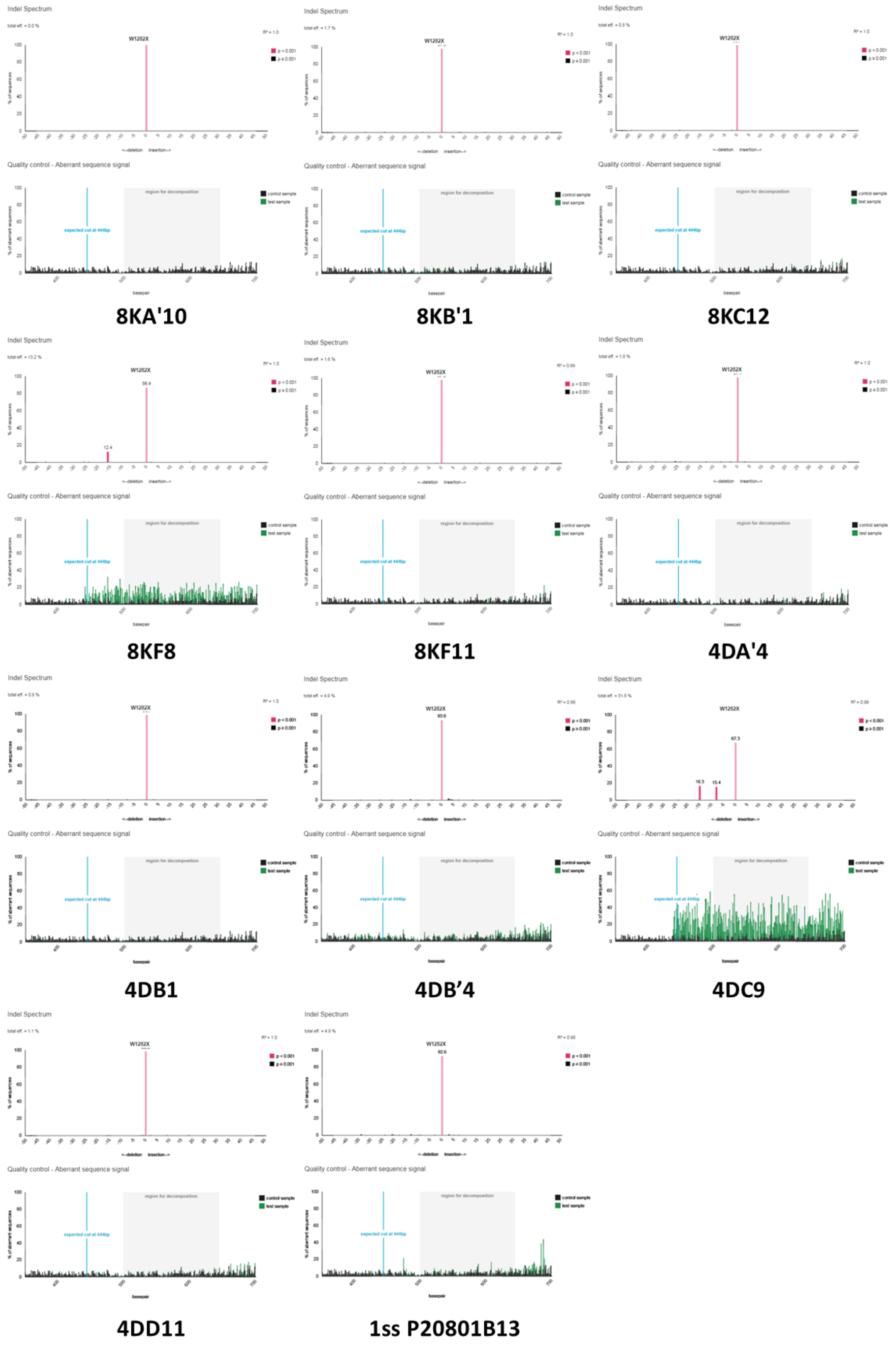

Indel Spectrum
W1282X
Quality control - Aberrant sequence signal
region for decomposition
expected cut at 444bp
control sample
test sample
8KA'10
8KB'1
8KC12
8KF8
8KF11
4DA'4
4DB1
4DB'4
4DC9
4DD11
1ss P20801B13

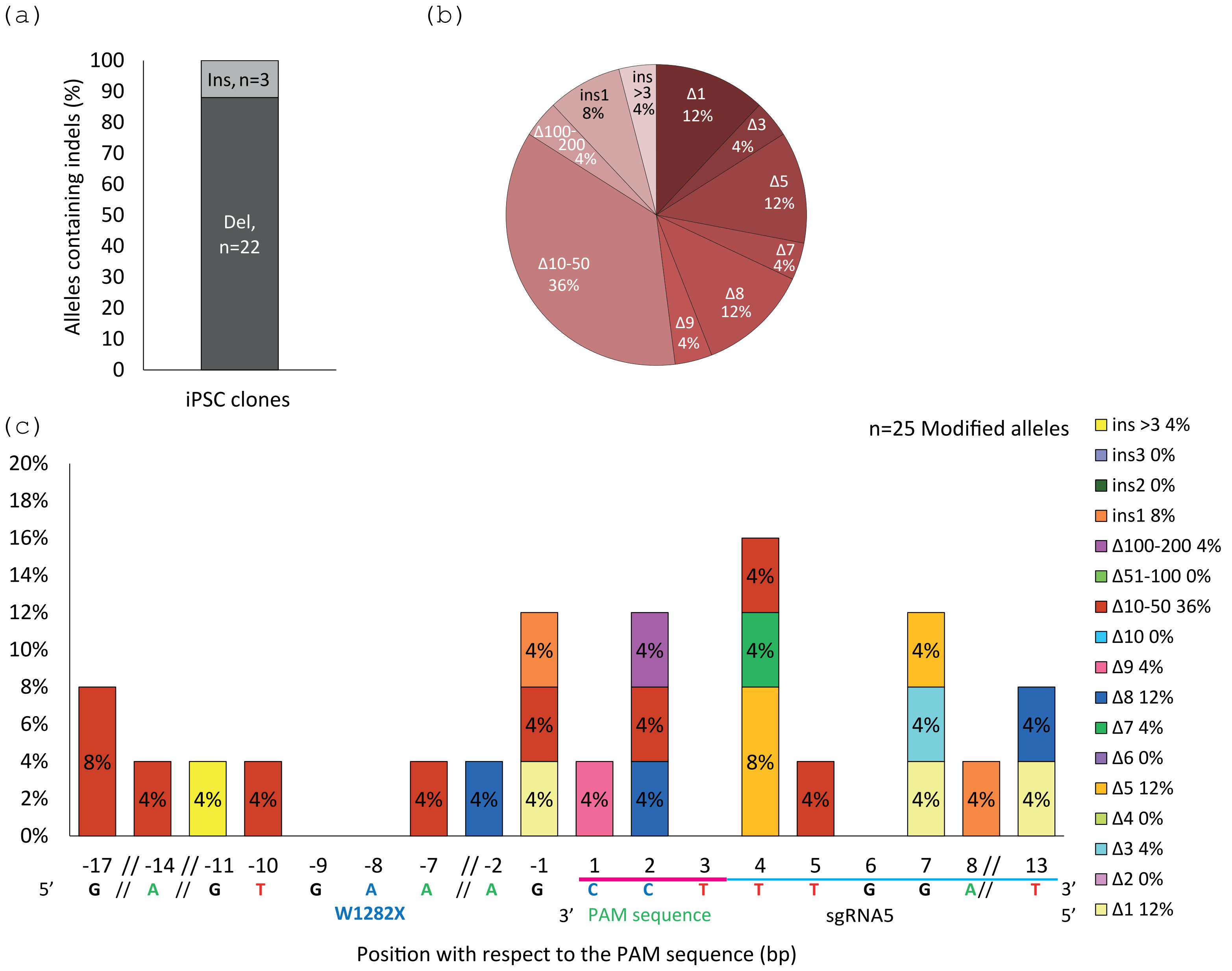

(a)
Alleles containing indels (%)
100
90
80
70
60
50
40
30
20
10
0
Ins, n=3
Del, n=22
iPSC clones
(b)
ins >3 4%
Δ1 12%
Δ3 4%
Δ5 12%
Δ7 4%
Δ8 12%
Δ9 4%
Δ10-50 36%
Δ100-200 4%
ins1 8%
(c)
n=25 Modified alleles
20%
18%
16%
14%
12%
10%
8%
6%
4%
2%
0%
-17 // -14 // -11 -10 -9 -8 -7 // -2 -1 1 2 3 4 5 6 7 8 // 13
5' G // A // G T G A A // A G C C T T T G G A// T 3'
W1282X
3' PAM sequence
sgRNA5
5'
Position with respect to the PAM sequence (bp)
ins >3 4%
ins3 0%
ins2 0%
ins1 8%
Δ100-200 4%
Δ51-100 0%
Δ10-50 36%
Δ10 0%
Δ9 4%
Δ8 12%
Δ7 4%
Δ6 0%
Δ5 12%
Δ4 0%
Δ3 4%
Δ2 0%
Δ1 12%

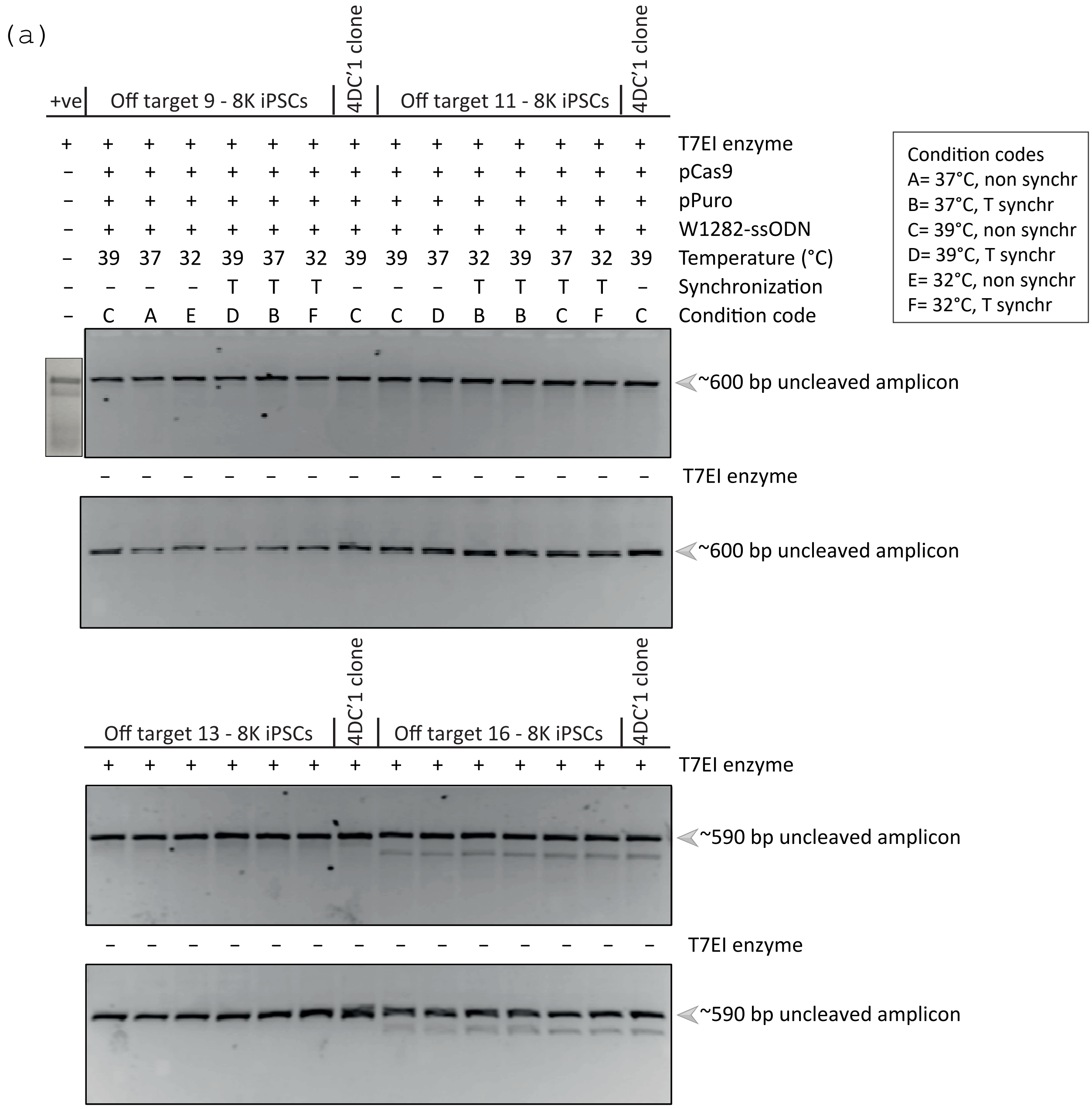
(a)
+ve
Off target 9 - 8K iPSCs
4DC'1 clone
Off target 11 - 8K iPSCs
4DC'1 clone
T7EI enzyme
pCas9
pPuro
W1282-ssODN
Temperature (°C)
Synchronization
Condition code
+ + + + + + + + + + + + + + +
– + + + + + + + + + + + + + +
– + + + + + + + + + + + + + +
– + + + + + + + + + + + + + +
– 39 37 32 39 37 32 39 39 37 32 39 37 32 39
– – – – T T T – – – T T T T –
– C A E D B F C C D B B C F C
Condition codes
A= 37°C, non synchr
B= 37°C, T synchr
C= 39°C, non synchr
D= 39°C, T synchr
E= 32°C, non synchr
F= 32°C, T synchr
~600 bp uncleaved amplicon
– – – – – – – – – – – – – –
T7EI enzyme
~600 bp uncleaved amplicon
Off target 13 - 8K iPSCs
4DC'1 clone
Off target 16 - 8K iPSCs
4DC'1 clone
+ + + + + + + + + + + + + +
T7EI enzyme
~590 bp uncleaved amplicon
– – – – – – – – – – – – – –
T7EI enzyme
~590 bp uncleaved amplicon

(b)

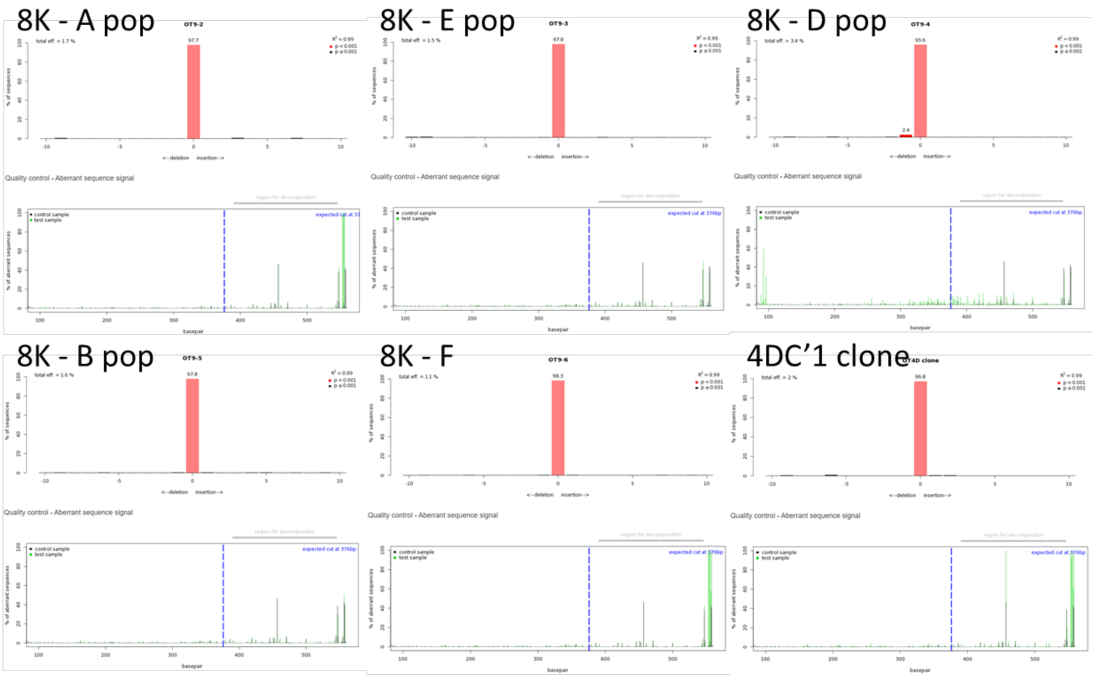
8K - A pop
8K - E pop
8K - D pop
Quality control - Aberrant sequence signal
Quality control - Aberrant sequence signal
Quality control - Aberrant sequence signal
8K - B pop
8K - F
4DC'1 clone
Quality control - Aberrant sequence signal
Quality control - Aberrant sequence signal
Quality control - Aberrant sequence signal

(c)

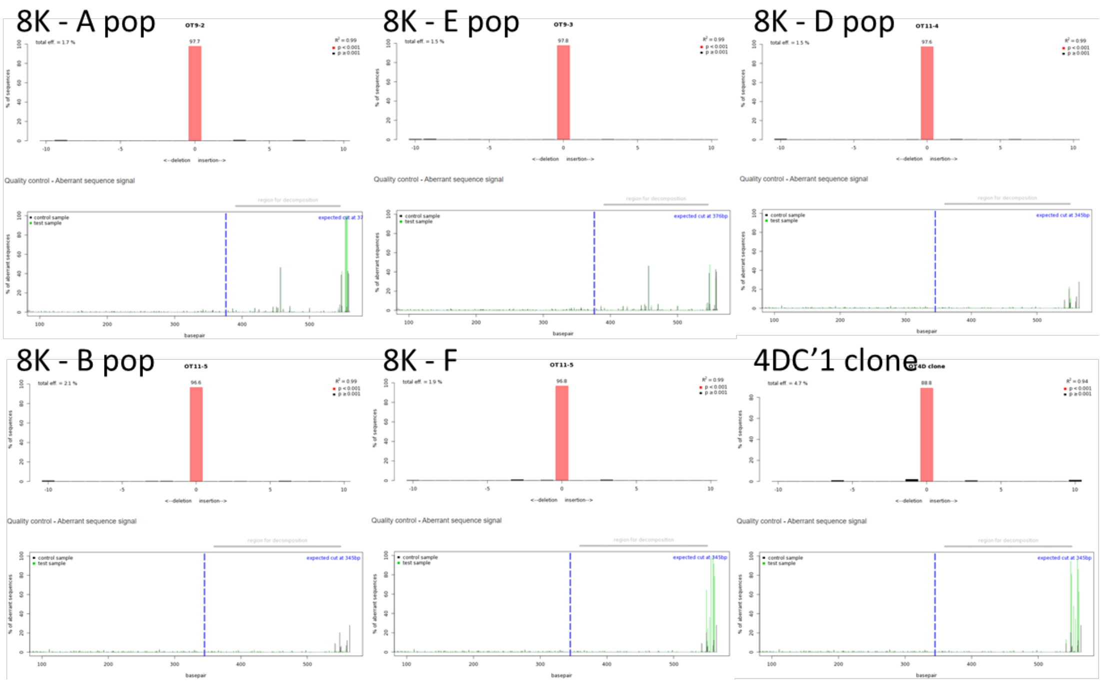
8K - A pop
8K - E pop
8K - D pop
Quality control - Aberrant sequence signal
Quality control - Aberrant sequence signal
Quality control - Aberrant sequence signal
8K - B pop
8K - F
4DC'1 clone
Quality control - Aberrant sequence signal
Quality control - Aberrant sequence signal
Quality control - Aberrant sequence signal

(d)

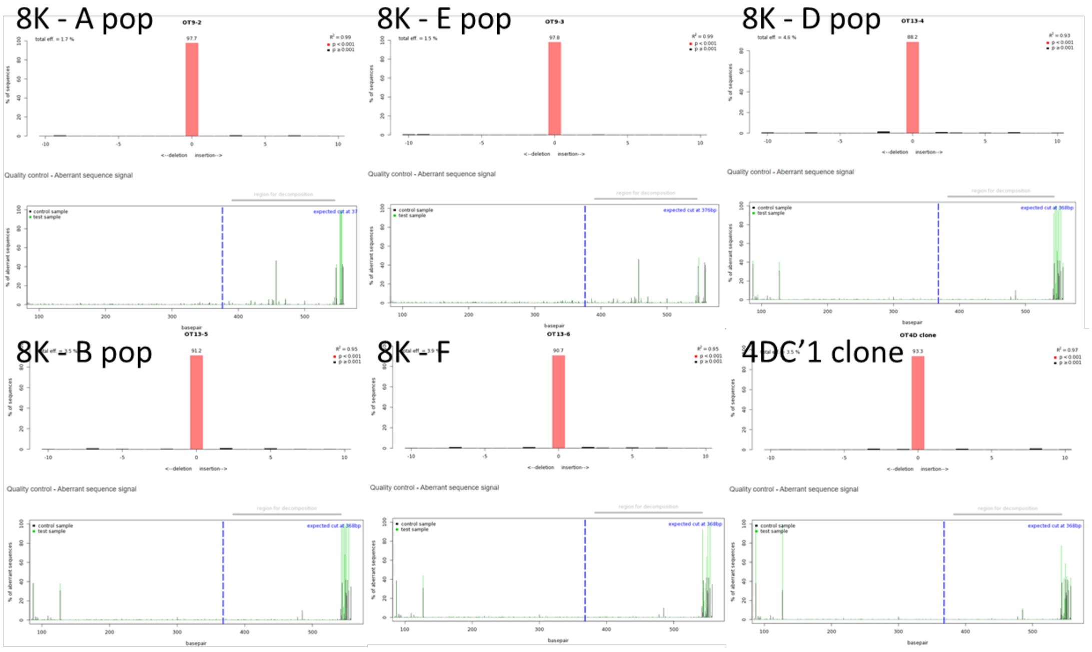
8K - A pop
OT9-2
8K - E pop
OT9-3
8K - D pop
OT13-4
8K - B pop
OT13-5
8K - F
OT13-6
4DC'1 clone
OT4D clone
Quality control - Aberrant sequence signal

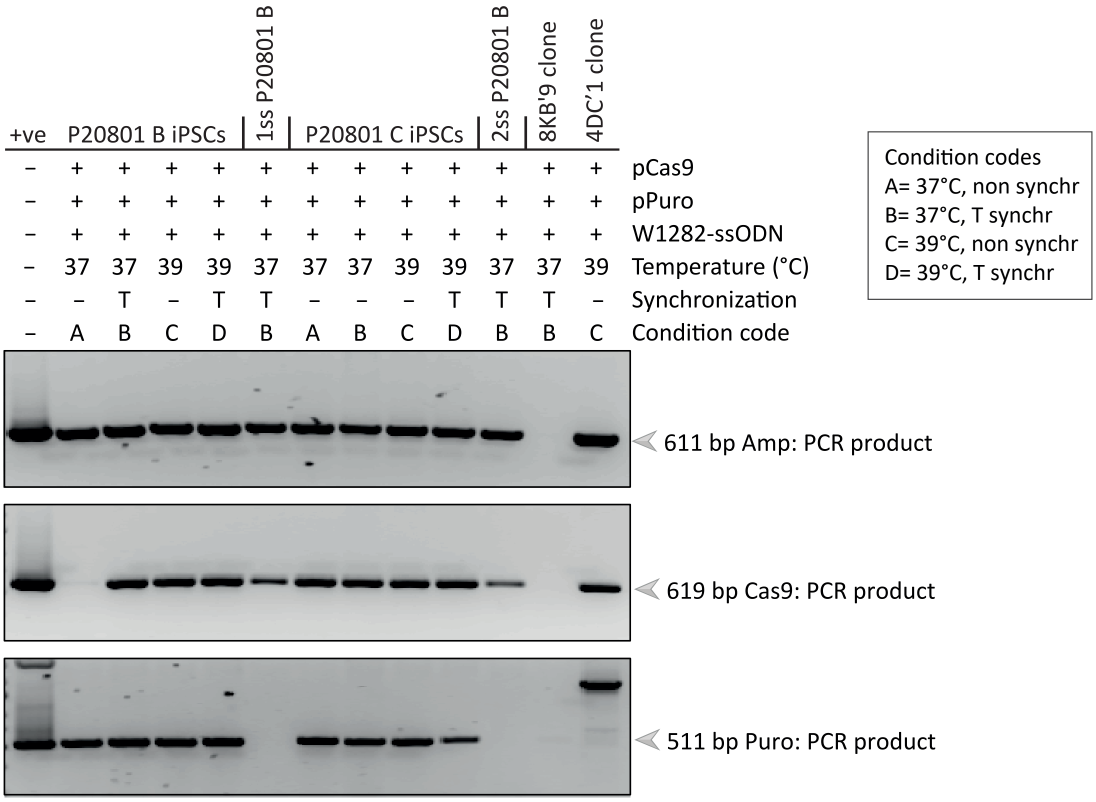

+ve
P20801 B iPSCs
1ss P20801 B
P20801 C iPSCs
2ss P20801 B
8KB'9 clone
4DC'1 clone
pCas9
pPuro
W1282-ssODN
Temperature (°C)
Synchronization
Condition code
611 bp Amp: PCR product
619 bp Cas9: PCR product
511 bp Puro: PCR product
Condition codes
A= 37°C, non synchr
B= 37°C, T synchr
C= 39°C, non synchr
D= 39°C, T synchr

Figure S1. Efficient transfection of CA1 cells achieved by nucleofection: (**a**) Nucleofection was performed in EDTA CA1 cells with 2.5 μg eGFP plasmid, resuspended in Ingenio® electroporation solution, by A-012 (green), A-013 (orange), A-023 (blue), A-027 (yellow) and B-016 (purple) programs. EDTA CA1 nucleofected cultures were assessed, 40 h after transfection, by flow cytometry (dot plots gates were set up at 0.1% threshold based on untransfected cells overlapped in grey, to calculate the transfection efficiency achieved by each program in purple, expressed as percentage); (**b**) Efficient transfection of CA1 cells upon nucleofection. A-023 and B-016 nucleofection programs were used in combination with the Ingenio® electroporation solution (Ingenio), human Stem Cell solution 1 (hSCS1) or human Stem Cell solution 2 (hSCS2). 1.45 x 10^6 CA1 cells were nucleofected with 2.5 μg eGFP plasmid per reaction. Transfection efficiency was quantified by flow cytometry as previously described in (A) for each set of conditions, 40 hours after nucleofection. Percentage of eGFP positive cells is shown by the bar graph, n=1 per set of conditions; (**c**) Transfection efficiency of EDTA CA1 and CA1 cells upon nucleofection of eGFP in Ingenio® electroporation solution using the B-016 program. Note that all cells were dissociated into single cells prior to nucleofection, independently of the passaging method. Transfection efficiency was assessed as described in Figure S1a legend. Each data point (n=9, n=15 biological replicates for EDTA and SC CA1 cells, respectively) plotted as circles. The centre lines show the medians; box limits indicate the quartiles; Tukey-whiskers extend 1.5 times the quartiles; crosses represent sample means. A significant difference was found in the transfection efficiency of EDTA CA1 and CA1 cells (nonparametric Mann-Whitney U test).

Figure S2. Nucleofected EDTA CA1 and CA1 cultures showed high expression of the pluripotency marker TRA-1-60: (**a**) The expression of the pluripotency marker TRA-1-60 was assessed by flow cytometry using as controls untransfected-unstained CA1 cells, untransfected-stained CA1 cells, as well as unstained CA1 cells-nucleofected with eGFP (see Figure S1c legend for further details), to set up the flow cytometry gates at 0.1% threshold; (**b**) Representative transfected EDTA and CA1 cells assessed for TRA-1-60 expression by flow cytometry, 40 hours post-nucleofection incubated under normothermia or hypothermia; (**c**) TRA-1-60 expression of untransfected CA1 (control), nucleofected EDTA and CA1 cells (incubated under normothermia or hypothermia) is represented as mean ±SEM (n=2 biological replicates for control and n=6 for nucleofected samples. No significant difference was found in the expression of TRA-1-60 pluripotency marker (nonparametric Kruskal-Wallis test).

Figure S3. The type and location of the indels identified in the CA1 clones: (**a**) TALEN-induced indels were identified in a total of 26 alleles from the sequenced CA1 clones. Most indels corresponded to deletions (black), although some insertions were also observed (grey); (**b**) Frequency of each type of deletions and insertions, ranging from 1 to 50 bp, shown as percentages of the 26 alleles containing indels; (**c**) Distribution of indels was mostly found within the spacer sequence, shown as frequency of each type of modification observed at the indicated positions. Total percentage of each type of modification is displayed on the right of each category.

Figure S4. Efficient transfection of 8K, 4D and P20801 iPSCs achieved by nucleofection: (**a**) Nucleofection of iPSC lines (3 x 10^6 cells per reaction) was performed with 2.5 μg eGFP plasmid, resuspended in human Stem Cell solution 1 (hSCS1), using B-016 program and incubated at 37°C and 32°C. Cell density and transfection efficiency were qualitatively assessed for each iPSC line and condition by phase-contrast and fluorescence microscopy, 40 hours after nucleofection. Scale bars, 100 μm; (**b**) Transfection efficiency was then quantified for each condition by flow cytometry, by setting up dot plots gates at 0.1% threshold based on untransfected cells, and eGFP positive cells were expressed as percentage, n=1 per condition.

Figure S5. 4D and P20801 nucleofected populations: (**a–b**) Phase-contrast microscopy images of the non- and S phase synchronised nucleofected (a) 4D and (b) P20801 iPSC populations. Procedure was performed as described in Figure 5a legend; 3 x 10^6 cells were nucleofected with 5 μg pCas9, 2 μg pPuro and 3.5 μl W1282-ssODN, resuspended in medium containing 10 μM Y-27632, and incubated for 2 days at 37°C, 39°C and 32°C. Then, transient puromycin selection was performed for (a) 4D iPSC populations

1 day after nucleofection (0.7 μg/mL for 1 day and 1 μg/mL for 1 day) and (b) for P20801 iPSC populations 2 days after nucleofection (1 μg/mL for 2 days). Scale bars, 100 μm.

Figure S6. TIDE analysis of all the chromatograms obtained by Sanger sequencing of the (**a**) 8K, (**b**) 4D, (**c**) P20801, and (**d**) P20801B frozen/thawed iPSC clones. Additionally, chromatograms obtained from clones that were considered (**e**) possibly positive and negative (**f**) were also assessed by TIDE for quality control purposes. Samples indicated with (*) were re-isolated and subclones were also sequenced and assessed by TIDE analysis (data not shown).

Figure S7. The type and location of the indels identified in the iPSC clones: (**a**) CRISPR-Cas9-mediated DSB induced indels identified in a total of 25 alleles from the 8K, 4D or P20801 iPSC clones. Most indels corresponded to deletions (black), although some insertions were also observed (grey); (**b**) Frequency of each type of deletions and insertions, ranged from 1 to 200 bp, shown as percentages of the 25 alleles containing indels; (**c**) Distribution of indels was mostly found around the PAM sequence, shown as frequency of each type of modification observed at the indicated positions. Total percentage of each type of modification is displayed on the right of each category.

Figure S8. Off-target analysis of 8K iPSC populations and a representative 4D iPSC clone: (**a**) T7EI assay analysis of the off-target 9, off-target 11, off-target 13, off-target 16, with and without the addition of T7EI enzyme. A sample confirmed to contain indels was used as control (+ve). TIDE analysis of the Sanger sequencing chromatograms of the amplified PCR product for each 8K iPSC population and 4DC'1 clone compared with 8K C population for (**b**) off-target 9, (**c**) 11 and (**d**) 13.

Figure S9. Random plasmid integration analysis of P20801 B, thawed 1ss P20801, P20801 C, thawed 2ss P20801 iPSC populations, representative 8KB'9 and 4DC'1 clones. Plasmids containing an ampicillin resistant cassette, the Cas9 encoding sequence, and a puromycin resistant cassette were used as positive controls (+ve). Amplification of 611 bp, 619 bp and 511 bp PCR products, respectively, suggested a potential plasmid integration or more likely the detection of remaining plasmids in the cells in the case of the iPSC populations.

Table S1. Genotyping of 8K, 4D and P20801 iPSC clones. Column 'clone': clones were colour-coded as results from AS-PCR W1282 (expected positive in red, possibly positive in blue, expected negative in bold black). Column 'ss': number of times that the populations, from which clones were isolated, were SC dissociated between nucleofection and clonal isolation; f indicates freezing using GCDR, non ss indicated as (ns). Column 'sequence': only shown the sequences identified at the highest percentages. It was indicated: A, G, or R (when A/G was identified at 50%) at W1282 position (green); PAM site CTT, CTT, or Y (when C/T was identified at 50%) (green); sgRNA binding site (underlined). Deletions (- - -) and insertions (orange) were also indicated. Column 'modified alleles': indicates how many alleles were modified in clones shown pure at the time of analysis; 'not pure' indicated otherwise, '0' was indicated when proportion of modified cells could not be obtained due to impure clones. Column 'modification (visually)': modifications visually identified from the chromatograms. Location of modifications are annotated as 'at x' position downstream (5'→ 3') from the PAM site. When several modifications were identified in the same sample, it was indicated as following: modifications in different cells of an impure clone and modifications in a pure clone, but it could not be determined whether they were present in the same or different allele was indicated (/). Modifications contained in the same allele (-) and modifications contained in different alleles (&) of the same pure clone. In cases of impure clones, indicated modifications were deduced from overlapped sequences and/or from sequencing analysis of several re-isolated and analysed subclones. Column 'modification (TIDE)': modifications obtained by TIDE analysis of chromatograms, and their frequencies in the samples indicated as a percentage of the total population. When modifications were not quantified but shown by the quality control graph indicated as 'QC confirmed'. Information was colour-coded based on: whether the results provided by visual analysis were confirmed (green), not detected (red), or new information was provided, not detected previously by visual analysis (blue).

Clone	ss	Sequence	Modified alleles	Modification (visually)	Modification (TIDE)
W1282X		TGCAACAGTGAAGGAAAGCCTTTGGAGTGATACCACAGgt		Located modifications	Indels present > 2%
8KA2	2	TGCAACAGTGAAGGAAAGCYTTTGGAGTGATACCACAGgt	1	het mutPAM	QC confirmed
8KA'8	2	TGCAACAGTGAAGGAAAGCCTT----------------gt	1	het del16 at5	51.4% del16
8KA'10	2	TGCAACAGTGAAGGAAAGCCTTTGGAGTGATACCACAGgt	0	-	Control for TIDE
8KB'1	2	TGCAACAGTGAAGGAAAGCCTTTGGAGTGATACCACAGgt	0	-	QC confirmed
8KB5	2	TGCAACAGTGAAGGAAAG---------TGATACCACAGgt	not pure	del9 at1	29.7% del9/27.3% del13/7.7% del1
8KB'9	2	TGCAACAGTGGAGGAAAGCTTTTGGAGTGATACCACAGgt	2	hom correct-mutPAM	QC confirmed
8KB'16	2	TGCAACAGTGAAGGAAAGCCTTTGGAGTGATACCACAGgt	0	-	QC confirmed
8KC12	2	TGCAACAGTGAAGGAAAGCCTTTGGAGTGATACCACAGgt	0	-	QC confirmed
8KC13	2	TGCAACAGTGAAGGAAAGCCTTTG-----ATACCACAGgt	not pure	mutPAM/del5 at7	- /3.2% del5
8KD6	2	TGCAACAGTGAAGGAAAGCCTTTG-AGTGATACCACAGgt	not pure	het correct/mutPAM/del1 at7	QC confirmed/19.5% del1
8KF2	2	TGCAACAGTGRAGGAAA-----------------------	2	het correct-mutPAM & del29 at-1	QC confirmed/53.3% del29
8KF8	2	TGCAACAGTGAAGGAAAGCCT----------------Ggt	not pure	del16 at4	12.4% del16
8KF11	2	TGCAACAGTGAAGGAAAGCCTTTGGAGTGATACCACAGgt	0	-	QC confirmed
8KF18	2	TGCAACAGTGAAGGAAAGCCTTTGGAGTGATACCACAGgt	0	-	QC confirmed
8KF19	2	TGCAACAGTGRAGGAAAGCYT-----GTGATACCACAGgt	1 or 2	het correct/mutPAM/del5 at4	QC confirmed/52.5% del5
4DA'4	2	TGCAACAGTGAAGGAAAGCCTTTGGAGTGATACCACAGgt	0	-	QC confirmed
4DA'9	2	TGCA---------------------------------Ggt	not pure	del33 at-14	3.1% del33
4DB1	2	TGCAACAGTGAAGGAAAGCCTTTGGAGTGATACCACAGgt	0	-	QC confirmed
4DB2	2	TGCAACAGTGAAGGAAAGC---------------------	2	het mutPAM & del154 at2	- /QC indicates a change
4DB'4	2	TGCAACAGTGAAGGAAAGCCTTTGGAGTGATACCACAGgt	0	-	QC confirmed
4DB'8	2	TGCAACAG------------------------------gt	2	het correct-mutPAM & del30 at-10	- /52.5% del30
4DB'17	2	TGCAACAGTGRAGGAAAGCYT-------GATACCACAGgt	1 or 2	het correct/mutPAM/del7 at4	QC confirmed/41% del7
4DC1	2	TGCAACAGTGAAGGAAAGCCTTTGGAGTGATACCACAGgt	not pure	correct/mutPAM	QC confirmed
4DC'1	2	TGCAACAGTGRAGGAAAGCYTTTGGAGTGATACCACAGgt	1 or 2	het correct/mutPAM	QC confirmed
4DC9	2	TGCAACAGTGAAGGAAAGC--------------------t	not pure	del15 at2/del8 at13	16.3% del15/15.4% del8
4DD'3	2	TGCAACAGTGAAGGAAAGCCTTTGGAGTGATACCACAGgt	0	-	QC confirmed
4DD11	2	TGCAACAGTGAAGGAAAGCCTTTGGAGTGATACCACAGgt	0	-	QC confirmed
P20801A1	ns	TGCAACAGTGAAGGAAAGCCTTTGGGAGTGATACCACAGgt	1 or 2	het mutPAM/ins1 at8	QC confirmed/45.8% ins1(59.5%G)
P20801A2	ns	TGCAACAGTGAAGGAAAGCCTTTGGAGTGATACCACAGgt	not pure	correct/mutPAM	- /2.5% del32
P20801B4	ns	TGCAACACTGAAGGAGAAATCCAGATCGATGGTGT----TGGGATTCAATAACTTTGCAACAGTGGAGGAAAGCTTTTGGAGTGATACCACAGgt	2	hom correct-mutPAM & ins55-4=51 at-11	- /QC indicates a change
P20801B7	ns	TGCAACAGTGGAGGAAAGCTTTTGGAGTGATACCACAGgt	2	hom correct-mutPAM	QC confirmed
P20801C5	1	TGCAACAGTGGAGGAAAGCTTTTGGAGTGATACCACAGgt	2	hom correct-mutPAM	QC confirmed
P20801C6	1	TGCAACAGTGGAGGAAAGCTTTTGGAGTGA-ACCACAGgt	2	hom correct-mutPAM-del1 at13	QC confirmed/96.9% del1
P20801C8	1	TGCAACAGTGAAGGAAAGCCTTTGGAGTGATACCACAGgt	0	-	QC confirmed
P20801C9	1	TGCAACAGTGAAGGAAAGCCTTTGGAGTGATACCACAGgt	not pure	-	3.1% del4
P20801D10	1	TGCAACAGTGAAGGAAAGC--------TGATACCACAGgt	2	hom del8 at2	96.7% del8

**1ss P20801B3**	f	TGCAACAGTGRAGGAAAGCTTTTGGAGTGATACCACAGgt	not pure	het correct-hom mutPAM	QC confirmed/3.7% del31
**1ss P20801B12**	f	T-------------------------------CCACAGgt	not pure	del31 at-17	16.5% del31
**1ss P20801B13**	f	TGCAACAGTGAAGGAAAGCCTTTGGAGTGATACCACAGgt	0	-	QC confirmed
**2ss P20801B3**	1,f	T-------------------------------CCACAGgt	not pure	del31 at-17	29.4%del15/25.3%del14/ 9.1% del31/7.8% del1
**2ss P20801B4**	1,f	TGCAACAGTGRAGGAAAGCTTTTGGAGTGATACCACAGgt	not pure	hom correct-mutPAM	- /QC indicates a change
**2ss P20801B5**	1,f	TGCAACAGTGAAGGAAAGCYTTTG-AGTGATACCACAGgt	not pure	hom correct-mutPAM	QC confirmed/19.1% del1
**2ss P20801B8**	1,f	TGCAACAGTGAAGGAA--------GAGTGATACCACAGgt	not pure	del8 at-2	41.3% del8/24.2% ins1
**2ss P20801B9**	1,f	TGCAACAGTGAAGGAAARCCT------TGATACCACAGgt	not pure	G>A at-1/del5 at4/del3 at7	- /56.6% del5/21.4% del3
**2ss P20801B10**	1,f	TGCAACAGTGA-------------------TACCACAGgt	not pure	del19 at-7	25.1% del2/22.8% del19

Table S2. Summary of the number of predicted potential off-targets obtained by Cas-OFFinder (http://www.rgenome.net/).

Target Sequence	Bulge Type	Mismatch	Bulge Size	Number of Found Targets	*In vitro* tests performed
CACCTGTGGTATCACTCCAANGG	DNA	1	1	1	See below
CACCTGTGGTATCACTCCAANGG	DNA	2	1	8	See below
CACCTGTGGTATCACTCCAANGG	DNA	3	1	247	
CACCTGTGGTATCACTCCAANGG	DNA	4	1	3549	
CACCTGTGGTATCACTCCAANGG	DNA	0	2	1	See below
CACCTGTGGTATCACTCCAANGG	DNA	1	2	2	See below
CACCTGTGGTATCACTCCAANGG	DNA	2	2	2	See below
CACCTGTGGTATCACTCCAANGG	DNA	3	2	96	
CACCTGTGGTATCACTCCAANGG	DNA	4	2	1650	

Table S3. Details of the top predicted potential off-targets obtained by Cas-OFFinder.

Off-target	Bulge Type	Target	Chromosome	Position	Direction	Mismatches	Bulge Size	*In vitro* tests performed
0	X	crRNA: CACCTGTGGTATCACTCCAANGG<br>DNA: CACCTGTGGTATCACTCCAAAGG	chr7	117642573	-	0	0	on target
1	DNA	crRNA: C-ACCTGTGGTATCACTCCAANGG<br>DNA: tCACCTGTGGTATCACTCCAAAGG	chr7	117642573	-	1	1	Not possible
2	DNA	crRNA: C--ACCTGTGGTATCACTCCAANGG<br>DNA: CTCACCTGTGGTATCACTCCAAAGG	chr7	117642573	-	0	2	Not possible
5	DNA	crRNA: CA--CCTGTGGTATCACTCCAANGG<br>DNA: CtCACCTGTGGTATCACTCCAAAGG	chr7	117642573	-	1	2	Not possible
6	DNA	crRNA: CAC--CTGTGGTATCACTCCAANGG<br>DNA: CtCACCTGTGGTATCACTCCAAAGG	chr7	117642573	-	1	2	Not possible
7	DNA	crRNA: CA-CCTGTGGTATCACTCCAANGG<br>DNA: tcACCTGTGGTATCACTCCAAAGG	chr7	117642573	-	2	1	Not possible
8	DNA	crRNA: CACC--TGTGGTATCACTCCAANGG<br>DNA: CtCaCCTGTGGTATCACTCCAAAGG	chr7	117642573	-	2	2	Not possible
9	DNA	crRNA: CACCTGTGGTATCA-CTCCAANGG<br>DNA: CACCTGTGGctTCACCTCCAAGGG<br>TIDEanalysis: CACCTGTGGctTCACCTCCA	chr2	241721184	-	2	1	T7, Sanger seq
10	DNA	crRNA: CACCTGTGGTATCAC-TCCAANGG<br>DNA: CACCTGTGGctTCACCTCCAAGGG	chr2	241721184	-	2	1	same as OT9
11	DNA	crRNA: CACCTGTGGTA-TCACTCCAANGG<br>DNA: CACgTGTGGTATTCAaTCCAACGG<br>TIDEanalysis: CACgTGTGGTATTCAaTCCA	chr16	66081948	-	2	1	T7, Sanger seq
12	DNA	crRNA: CACCTGTGGTAT-CACTCCAANGG<br>DNA: CACgTGTGGTATTCAaTCCAACGG	chr16	66081948	-	2	1	same as OT11
13	DNA	crRNA: CACCTGTGGTATC-ACTCCAANGG<br>DNA: CACCTGTGGTATCAAaTCCAgAGG<br>TIDEanalysis: CACCTGTGGTATCAAaTCCA	chr3	64367349	+	2	1	T7, Sanger seq
14	DNA	crRNA: CACCTGTGGTATCA-CTCCAANGG<br>DNA: CACCTGTGGTATCAAaTCCAgAGG	chr3	64367349	+	2	1	same as OT13
15	DNA	crRNA: CACCTGTGGTATCAC-TCCAANGG<br>DNA: CACCTGTGGTATCAaATCCAgAGG	chr3	64367349	+	2	1	same as OT13
16	DNA	crRNA: CACCTGT--GGTATCACTCCAANGG<br>DNA: CACgTGTTCGGTATCACTgCAATGG	chr2	11778003	-	2	2	T7

Not possible: the first eight identified off-targets in chromosome 7 are located within a few nucleotides of the on-target side, therefore, targeting of these sites would result in almost identical outcomes as compared with on-target activity, likely leading to the same desired effects as the on-target activity, thus, the *in vitro* assessment of these off-targets was not possible.

Table S4. Results of the Sanger sequencing analysis of the selected predicted CRISPR-Cas9 off-targets.

Off-target	Sample	Sequence	Modification
9	8K pop-C	TGAGAGGCCAGTGAGgCCTgCACGGTGGCCTCAGCTTC	-
	8K pop-A	TGAGAGGCCAGTGAGgCCTgCACGGTGGCCTCAGCTTC	-
	8K pop-E	TGAGAGGCCAGTGAGgCCTgCACGGTGGCCTCAGCTTC	-
	8K pop-D	TGAGAGGCCAGTGAG g/a CCT g/a CACGGTGGCCTCAGCTTC	heterozygous 1bp substitutions at 95, 99 G<A G<A (2.4%)
	8K pop-B	TGAGAGGCCAGTGAGgCCTgCACGGTGGCCTCAGCTTC	-
	8K pop-F	TGAGAGGCCAGTGAGgCCTgCACGGTGGCCTCAGCTTC	-
	4DC'1 clone	TGAGAGGCCAGTGAGgCCTgCACGGTGGCCTCAGCTTC	SNP detected: TC heterozygous at 458 8K, CC homozygous 4D iPSC line
11	8K pop-C	TGTAGAGTAAACCATTTTTAtTGGTGTTGAGAGGAGTGGT	-
	8K pop-A	TGTAGAGTAAACCATTTTTAtTGGTGTTGAGAGGAGTGGT	-
	8K pop-E	TGTAGAGTAAACCATTTTTAtTGGTGTTGAGAGGAGTGGT	-
	8K pop-D	TGTAGAGTAAACCATTTTTAtTGGTGTTGAGAGGAGTGGT	-
	8K pop-B	TGTAGAGTAAACCATTTTTAtTGGTGTTGAGAGGAGTGGT	-
	8K pop-F	TGTAGAGTAAACCATTTTTAtTGGTGTTGAGAGGAGTGGT	-
	4DC'1 clone	TGTAGAGTAAACCATTTTTA t/c TGGTGTTGAGAGGAGTGGT	SNP detected: TT homozygous at 79 8K, TC heterozygous 4D iPSC line
13	8K pop-C	AATTTTAGATGGAGTTTTAG a/g GGTGTACTCCTGTAAGCTT	-
	8K pop-A	AATTTTAGATGGAGTTTTAG a/g GGTGTACTCCTGTAAGCTT	-
	8K pop-E	AATTTTAGATGGAGTTTTAG a/g GGTGTACTCCTGTAAGCTT	-
	8K pop-D	AATTTTAGATGGAGTTTTAG a/g GGTGTACTCCTGTAAGCTT	-
	8K pop-B	AATTTTAGATGGAGTTTTAG a/g GGTGTACTCCTGTAAGCTT	-
	8K pop-F	AATTTTAGATGGAGTTTTAG a/g GGTGTACTCCTGTAAGCTT	-
	4DC'1 clone	AATTTTAGATGGAGTTTTAGgGGTGTACTCCTGTAAGCTT	SNP detected: AG heterozygous at 87 8K, GG homozygous 4D iPSC line

16	8K pop-C	ACATGGACGTGTGGGGGCCCACGTGCCTTCTTATAGGAC	
	8K pop-A	ACATGGACGTGTGGGGGCCCACGTGCCTTCTTATAGGAC	
	8K pop-E	ACATGGACGTGTGGGGGCCCACGTGCCTTCTTATAGGAC	
	8K pop-D	ACATGGACGTGTGGGGGCCCACGTGCCTTCTTATAGGAC	
	8K pop-B	ACATGGACGTGTGGGGGCCCACGTGCCTTCTTATAGGAC	
	8K pop-F	ACATGGACGTGTGGGGGCCCACGTGCCTTCTTATAGGAC	
	4DC'1 clone	ACATGGACGTGTGGGGGCCCcACGTGCCTTCTTATAGGAC	Insertion 1bp (c) at 532

Table S5. Primer list for PCR reactions and sequencing analysis. Note that primers in uppercase bind to exon regions, while lowercase primers bind to intron regions, and primers indicated as 'repeat' were used for several programs.

PCR name	Primer name	Primer sequence 5′ → 3′
**Ex11newT7 PCR**	Fw_newT7	TGATTATGGGAGAACTGGAGC
**Ex11newT7 PCR**	Rev_CFTR11	ccattcacagtagcttaccc
**Ex11newT7 Phire-PCR**	Fw_newT7	repeat
**Ex11newT7 Phire-PCR**	Rev_CFTR11	repeat
**ASPCR Nor**	Fw _Nor	GGCACCATTAAAGAAAATATCATCTT
**ASPCR Nor**	Rev _Nor	caatgctcattccattaggctata
**ASPCR ΔF508**	Fw _CTT	GGCACCATTAAAGAAAATATCATTGG
**ASPCR ΔF508**	Rev _CTT	gatcaatgctcattccattaggctata
**Ex23CFTR HiFi-PCR**	Fw_CFTR23	Gtgcatgaagacactctttgc
**Ex23CFTR HiFi-PCR**	Rev_CFTR23	Aacttggaggtcaggccact
**Ex23CFTR PCR**	Fw_CFTR23	repeat
**Ex23CFTR PCR**	Rev_CFTR23	repeat
**ASPCR W1282**	Fw_W1282	TGCAACAGTGGAGGAAAGCT
**ASPCR W1282**	Rev_CFTR23	repeat
**Off-target 9**	Fw_OT9	GGGTCACCCTGGCCT
**Off-target 9**	Rev_OT9	GACAGGGGTTTCTCTTTAGAGC
**Off-target 11**	Fw_OT11	TTCATCAAGTCACTTGGTGGG
**Off-target 11**	Rev_OT11	GTGGGAAGGATTCAGGTGTG
**Off-target 13**	Fw_OT13	TCTGGCTGTAACAAACAGGTAC
**Off-target 13**	Rev_OT13	TCCATGTAAGCATCAGGTGC
**Off-target 16**	Fw_OT16	CGGGCATAGGCAGTTGG
**Off-target 16**	Rev_OT16	TGCCCTTTAGGGAGCAGT
**Amp**	Fw_Amp	CAGCGATCTGTCTATTTCGTTCA
**Amp**	Rev_Amp	GCTATGTGGCGCGGTATTAT
**Cas9**	Fw_Cas9	GTGGACGAGGTGGCCTA
**Cas9**	Rev_Cas9	GGTGCTCGTCGTATCTCTTG
**Puro**	Fw_Puro	gagtacaagcccacggtg
**Puro**	Rev_Puro	gctcgtagaaggggaggt
